# GATAD2B O-GlcNAcylation Regulates Breast Cancer Stem-like Potential and Drug Resistance

**DOI:** 10.3390/cells14060398

**Published:** 2025-03-08

**Authors:** Giang Le Minh, Jessica Merzy, Emily M. Esquea, Nusaiba N. Ahmed, Riley G. Young, Ryan J. Sharp, Tejsi T. Dhameliya, Bernice Agana, Mi-Hye Lee, Jennifer R. Bethard, Susana Comte-Walters, Lauren E. Ball, Mauricio J. Reginato

**Affiliations:** 1 Department of Biochemistry and Molecular Biology, Drexel University College of Medicine, Philadelphia, PA 19102, USA; gleminh91@gmail.com (G.L.M.);; 2 Department of Cell and Molecular Pharmacology and Experimental Therapeutics, Medical University of South Carolina, Charleston, SC 29425, USAballle@musc.edu (L.E.B.); 3 Translational Cellular Oncology Program, Sidney Kimmel Cancer Center, Thomas Jefferson University, Philadelphia, PA 19107, USA

**Keywords:** OGT, O-GlcNAc, cancer, cancer stem cell, signaling, NuRD, GATAD2B, chemoresistance

## Abstract

The growth of breast tumors is driven and controlled by a subpopulation of cancer cells resembling adult stem cells, which are called cancer stem-like cells (CSCs). In breast cancer, the function and maintenance of CSCs are influenced by protein O-GlcNAcylation and the enzyme responsible for this post-translational modification, O-GlcNAc transferase (OGT). However, the mechanism of CSCs regulation by OGT and O-GlcNAc cycling in breast cancer is still unclear. Analysis of the proteome and O-GlcNAcome, revealed GATAD2B, a component of the Nucleosome Remodeling and Deacetylase (NuRD) complex, as a substrate regulated by OGT. Reducing GATAD2B genetically impairs mammosphere formation, decreases expression of self-renewal factors and CSCs population. O-GlcNAcylation of GATAD2B at the C-terminus protects GATAD2B from ubiquitination and proteasomal degradation in breast cancer cells. We identify ITCH as a novel E3 ligase for GATAD2B and show that targeting ITCH genetically increases GATAD2B levels and increases CSCs phenotypes. Lastly, we show that overexpression of wild-type GATAD2B, but not the mutant lacking C-terminal O-GlcNAc sites, promotes mammosphere formation, expression of CSCs factors and drug resistance. Together, we identify a key role of GATAD2B and ITCH in regulating CSCs in breast cancer and GATAD2B O-GlcNAcylation as a mechanism regulating breast cancer stem-like populations and promoting chemoresistance.

## 1. Introduction

Breast cancer is a major health challenge affecting millions of women worldwide [1]. While advancements in breast cancer prognosis and treatment have made significant progress, the complexity of breast cancer outcomes is still influenced by factors such as tumor heterogeneity. Tumor cell heterogeneity is thought to be maintained and promoted by CSCs, a sub-population of cancer cells that are capable of self-renewing and differentiating [2,3,4]. CSCs are a subpopulation of cells within a tumor that possess characteristics similar to normal stem cells. They are capable of self-renewal, differentiation, and sustaining tumor growth. These cells play a critical role in cancer progression including metastasis, resistance to therapy, and recurrence [3,5,6]. Understanding the properties and functional activities of CSCs, as well as the regulatory mechanism of CSCs hold promise for developing novel therapeutic strategies and improving efficacy of breast cancer treatment [7].

Cancer cells are highly dependent on glycolysis to meet the demand for rapid proliferation and tumor growth [8]. However, beside the major influx of glucose being consumed in glycolysis, a small percentage of glucose entering cancer cells is used in the hexosamine biosynthesis pathway (HBP), which utilizes glucose and other products of major metabolic pathways to form UDP-N-acetylglucosamine (UDP-GlcNAc) [9]. UDP-GlcNAc serves as the substrate for N-linked and O-linked glycosylation of membrane and secreted proteins [10], and for modification of intracellular proteins in a process called O-GlcNAcylation [11]. This modification modulates protein stability, protein-protein interaction, protein phosphorylation and protein localization [12]. Both O-GlcNAcylation and its enzyme O-GlcNAc transferase (OGT) are highly expressed in many cancers, where it promotes tumor growth [13,14] and metastasis [15,16]. Importantly, recent studies have implicated the role of elevated OGT and O-GlcNAc in regulating CSCs phenotypes and tumor initiation [17]. However, the mechanism by which OGT and O-GlcNAc regulate CSCs in cancer is not clear.

The Nucleosome Remodeling and Deacetylase complex (NuRD complex) is a multi-protein complex that participates in regulation of gene expression by restructuring chromatin architecture and by deacetylation of histone. The NuRD complex plays a pivotal role in maintaining cellular homeostasis, regulating gene expression during development, differentiation, and DNA repair [18]. The complex is also critical for stem cell function and progenitor cells activities [19,20]. A number of components of the NuRD complex have been linked to cancer, including GATAD2B, a key scaffold-protein in the complex [21,22]. However, the function of GATAD2B in breast cancer has not yet been explored. Here, we show for the first time that GATAD2B is critical in maintaining and promoting CSCs phenotypes while O-GlcNAcylation of GATAD2B enhances its stability and protects from ITCH-mediated proteasomal degradation and contributes to cancer stem cell functions in breast cancer cells.

## 2. Materials and Methods

### 2.1. Cell Lines

Human TNBC cells MDA-MB-231 were purchased from the ATCC (American Type Culture Collection, Manassas, VA, USA), and were cultured in humidified condition with 5% CO_2_ in complete medium Dulbecco’s Modification Eagle’s Medium with 4.5 g/L glucose (DMEM, Genesee Scientific, El Cajon, CA, USA), supplemented with 10% fetal bovine serum (FBS, GeminiBio, West Sacramento, CA, USA), 1% L-Glutamine (Gibco/ThermoFisher Scientific, Waltham, MA, USA), 1% penicillin/streptomycin (Invitrogen, Carlsbad, CA, USA). TNBC cells SUM159 and patient-derived xenograft TNBC cells HCI-10 were received as a kind gift from Dr. Seagroves (University of Tennessee). SUM159 cells were maintained in DMEM/F12 medium with 10% FBS (Gemini), 1% pencicillin/streptomycin (Invitrogen), 1% L-Glutamine (Gibco) and 0.05% Hydrocortisone. HCI-10 cells were cultured in DMEM/F12 medium with 2% of low endotoxin FBS (Gibco), 1× penicillin/streptomycin (Gibco), 1× Insulin-transferrin-selenium, 5 ng/mL hEGF, 0.5 ng/mL chorela toxin, 0.3 μg/mL hydrocortisone, 5 nM 3,3′,5-triiodo-L-thyronine, 5 μM isoproterenol hydrochloride, 50 nM ethanolamine, 50 nM O-phosphorylethanolamine and 10 mM HEPES. HEK-293T cell was purchased from ATCC (American Type Culture Collection, Manassas, VA, USA) and was cultured in humidified condition at 37 °C with 5% CO_2_ using Dulbecco’s Modification Eagle’s Medium with 4.5 g/L glucose (DMEM, Genesee) with 10% FBS (Gemini), 1% L-Glutamine (Gibco/Life-Technologies), and 1% penicillin/streptomycin (Invitrogen). Quarterly testing was conducted to ensure that all the cells remained free from mycoplasma contamination (abmGood PCR Mycoplasma Detection Kit, Richmond, BC, Canada).

### 2.2. Generating Cells with GATAD2B or OGT Overexpression

To generate TNBC cells stably overexpressing GATAD2B or OGT, lentiviral transduction was performed as previously described [17] using the respective lentiviral vectors obtained from GeneCopoeia, Rockville, MD, USA (EX-E0581-Lv118-GATAD2B and EX-Z3428-Lv101-OGT). Construct containing GATAD2B S584/586/588/590A mutant was generated and obtained from GeneCopoeia based on the template of EX-E0581-Lv118-GATAD2B.

### 2.3. Transient Transfection of Mammalian Expression Constructs

Transient transfection was conducted in HEK293T cells, which were cultured in the condition described above. Transfection with PEI was performed as described before using 15 µg of DNA construct. pRK5-HA-Ub was purchased from Addgene, Watertown, MA, USA, from Dr. Dawson (John Hopkins University School of Medicine). Wild-type HA-tagged GATAD2B and mutant GATAD2B (S584/586/588/590A) was obtained from GeneCopoeia (EX-E0581-Lv118-GATAD2B). Protein expression was allowed to continue for 3 days after transfection, prior to further analysis.

### 2.4. RNA Interference

GATAD2B knockdown was performed using lentiviral vectors carrying shRNA constructs obtained from Sigma-Aldrich, St. Louis, MO, USA, (TRCN0000015315, TRCN0000015317). The sequences of used shRNA targeting GATAD2B are: 5′-CGCTCCATGCTTTCAAACTTT-3′ (shGATAD2B #1) and 5′-CAGGAAATTGAACAGCGATTA-3′ (shGATAD2B #2). ITCH knockdown was conducted using lentiviral shRNA constructs purchased from Sigma-Aldrich (TRCN0000002087, TRCN0000010680). The sequences of used shITCH constructs are: 5′-CCAGAAGTCAAGGTCAATTAA-3′ (shITCH #1) and 5′-CGAAGACGTTTGTGGGTGATT-3′ (shITCH #2). Control shRNA (plasmid 1864) was purchased from Addgene. OGT shRNA was obtained from Sigma-Aldrich. The sequence of used shRNA targeting OGT is: 5′-GCTGAGCAGTATTCCGAGAAACTCGAGTTTCTCGGAATACTGCTCAGC-3′ (shOGT). The sequence of used control shRNA is: 5′-CCTAAGGTTAAGTCGCC-CTCGCTCGAGCGAGGGCGACTTAACCTTAGG-3′. pLKO1-puro vectors carrying shRNA targeting GATAD2B, ITCH, OGT, or control shRNA were packaged into VSVG-pseudo-lentiviral particles using HEK-293T cells. Lentiviral transduction was conducted as described before to generate GATAD2B, ITCH or OGT knockdown.

CRISPR targeting GATAD2B was conducted using gRNA obtained from Sigma-Aldrich (CRISPRD HSPD0000102326) with the following sequence 5′-TCGCTTGAATCTGTTGAAG-3′. The control gRNA was also obtained from Sigma-Aldrich (CRISPRD NegativeControl1) with the following sequence 5′-CGCGATAGCGCGAATATATT-3′. All-in-one lentiviral vector LV01-U6-gRNA:ef1a-puro-2A-Cas9-2A-tGFP carrying gRNA targeting GATAD2B or control gRNA was packaged into VSVG-pseudo-lentiviral vector using HEK293T cells. Lentiviral transduction was conducted as described before.

### 2.5. Mammosphere Formation Assay

Breast cancer cells were collected and a fixed amount of cells (between 200–800 cells depending on mammosphere formation effeciency of specific cell line) was cultured in ultralow-attachment 96-well plate, pre-coated with 1.2% polyHEMA (Corning) as previously described [17]. Seeded cells were allowed to form in DMEM/F12 (Gibco) medium, supplemented with 1 mg/mL Pen/Strep (Gibco), 1× B27 (Invitrogen, 17504-044), 20 ng/mL EGF (Sigma-Aldrich) and 20 ng/mL bFGF (Gibco, PHG0024). Mammospheres were allowed to form in humidified condition with 5% CO_2_, at 37 °C for 5–7 days. The number of mammosphere greater than 50 µm was counted and mammosphere formation efficiency was determined using the following formula: (number of mammospheres/number of cells plated) * 100%.

For secondary mammosphere, breast cancer cells were allowed to grow in 6-well plate, coated with 1.2% polyHEMA in the same condition as described above. After 5–7 days, mammospheres were collected to prepare single-cell suspension. A fixed number of cells (200–800 cells) was then cultured in the mammosphere forming assay as described above. the same condition as described above. The efficiency of mammosphere formation was determined following the same formula as described above.

### 2.6. Flow Cytometry

ALDEFLOUR assay was performed using ALDEFLOUR assay kit purchased from STEMCELL Technologies, Vancouver, British Columbia, Canada, as per manufacturer instruction. In brief, breast cancer cells were harvested, washed in PBS twice, and resuspneded in ALDEFLOUR assay buffer at concentration of 2 × 10^5^ cells/mL. 1 mL of cell suspension was then rapidly mixed with 5 μL of activated provided substrate BAAA-DA and 500 μL of the mixture was immediately taken out and mixed with 5 μL of ALDGH-specific inhibitor-DEAB. Samples with DEAB and without DEAB were then incubated at 37 °C for 30 min, followed by centrifugation to remove exceeded BAAA-DA.

The cell pellet was resuspended in 500 μL of ALDEFLOUR assay buffer. Fluorescence signal generated from ALDH activity in the presence of BAAA-DA was detected by flow cytometry using the system Guava-easyCyte (MilliporeSigma, Burlington, MA, USA). The basal fluorescence signal was determined using sample with ALDH inhibitor DEAB. The percentage of ALDH-positive cells represents the population of cancer cells with high ALDH activity.

SOX2/Oct4-Response element GFP (SORE-GFP) reporter was received as a kind gift from Dr. Wakefield (NCI) [23]. Lentiviral vectors containing SORE-GFP construct were generated in HEK293T cells, and lentiviral transduction was conducted as described before. A construct containing SORE-GFP with minimal-CMV promoter also received from Dr. Wakefield (NCI) was used as a control. For SORE-GFP assay, breast cancer cells transduced with control or SORE-GFP construct were collected, washed in PBS, and counted. 2 × 10^5^ cells from each sample was pelleted and resuspended in 500 μL PBS. The fluorescence emission from GFP was assessed by flow cytometry using the system Guava-easyCyte (Millipore). Basal fluorescence signal was determined using the control sample with minimal CMV promoter.

### 2.7. Western Blotting

Breast cancer cells were harvested, centrifuged, and thoroughly washed with ice-cold PBS. Cells were then lysed in RIPA buffer containing 150 mM NaCl, 1% NP40, 0.5% Deoxycholate, 50 mM Tris-HCl (pH 8), 0.1% SDS, 10% glycerol, 5 mM EDTA, 20 mM NaF, and 1 mM Na_3_VO_4_, supplemented with protease inhibitors. After lysis, all lysates were centrifuged at 15,000 rpm for 20 min at 4 °C and supernatants were collected for immunoblot analysis. Protein concentration was determined using Bradford assay. 50–100 ug of total proteins was separated by SDS-PAGE and transferred to PVDF membrane with 0.45 µm pore size. The following primary antibodies were used to detected proteins of interest: Anti-OGT (Cell-Signaling, Danvers, MA, USA, cat #20438), Anti-O-GlcNAc (Sigma, cat #MABS1254), Anti-c-MYC (Novus Biologicals, Centennial, CO, USA, cat #NB600-335), Anti-Actin (Santa Cruz, Dallas, TX, USA, cat #sc-47778), Anti-NANOG (Cell-Signaling, cat #3580), Anti-SOX2 (Cell-Signaling, cat #3579), Anti-OCT4 (Cell-Signaling, cat #2750), Anti-GATAD2B (Cell-Signaling, cat #73098), anti-GATAD2A (Cell-Signaling, cat #17705), anti-MTA2 (Sigma, cat #HPA006214), anti-CHD4 (Cell-Signaling, cat #12011), anti-HDAC2 (Cell-Signaling, cat #57156), anti-RBBP4 (Cell-Signaling, cat #9067), anti-ITCH (Cell-Signaling, cat #12117).

### 2.8. Mass Spectrometry Analysis

Effect of Thiamet G on protein expression. MDA-MB-231 cells were grown in the presence or absence of Thiamet G (Sigma-Aldrich) at a final concentration of 1 µM for 24 h. Cells were washed three times with cold PBS prior to harvesting and lysed with freshly prepared lysis buffer (9 M urea, 50 mM Tris (pH 8), and 100 units/mL Pierce Universal Nuclease (Thermo Fisher Scientific, Waltham, MA, USA)). The lysates were sonicated (Fisherbrand Model 120 Sonic Dismembrator, Fisher Scientific) by applying 20 s on/off pulses for 3 cycles and centrifuged at 16,000× *g* for 15 min to pellet cell debris. Protein concentration was determined by bicinchoninic acid protein assay (Pierce BCA Protein Assay Kit, Thermo Scientific). Proteins were reduced with 1 mM 1,4-Dithiothreitol (DTT) (Thermo Scientific) for 30 min at 25 °C followed by alkylation with 5.5 mM iodoacetamide (IAA) (Thermo Scientific) for 15 min at 25 °C in the dark. The urea concentration was diluted to ~1.6 M with 50 mM ammonium bicarbonate (pH 8.0) prior to sequential digestion with Lys-C (1:50 enzyme: protein ratio, Wako Tokyo, Japan) for 3 h at 25 °C and trypsin (1:50 enzyme: protein ratio, Sigma) at 37 °C for 18 h. Acidified samples were desalted using C18 StageTips (Thermo Scientific SP301) and dried by vacuum centrifugation. Peptides were separated and analyzed on an EASY nLC 1200 System (Thermo Scientific) in-line with the Orbitrap Fusion Lumos Mass Spectrometer (Thermo Scientific) with instrument control software v. 4.2.28.14. Two µg of tryptic peptides were pressure loaded onto C18 reversed phase column (Acclaim PepMap RSLC, 75 µm × 50 cm (C18, 2 µm, 100 Å) ThermoFisher cat. #164536) using a gradient of 5% to 35% B in 180 min (Solvent A: 5% acetonitrile/0.1% formic acid; Solvent B: 80% acetonitrile/0.1% formic acid) at a flow rate of 300 nL/min. Mass spectra were acquired in data-dependent mode with a high resolution (60,000) FTMS survey scan, mass range of *m*/*z* 375–1575, followed by tandem mass spectra (MS/MS) of the most intense precursors with a cycle time of 3 s. The automatic gain control target value was 4.0 × 10^5^ for the survey MS scan. HCD fragmentation was performed with a precursor isolation window of 1.6 *m*/*z*, a maximum injection time of 50 ms, and collision energy of 35%. Monoisotopic-precursor selection was set to “peptide”. Precursors within 10 ppm mass tolerance were dynamically excluded from resequencing for 15 s. Precursor ions with charge states 2–5 were included. Data were searched against a forward and reversed, decoy human protein database downloaded from Uniprot on 4 February 2020 as well as a database of common cell culture contaminants using the MaxQuant (v 1.0.17.0) (Max Planck Institute). The search parameters allowed a maximum of two missed cleavages and a minimum peptide length of seven amino acids. Cysteine carbamidomethylation was set as a fixed modification while acetylation at protein N-termini and oxidation of methionines were set as variable modifications. Matching between runs was enabled. For identification an FDR < 0.01 was required at the PSM, peptide, and protein levels. Data were processed using Perseus v1.6.12.0 (Max Planck Institute). Proteins were filtered to remove matches to the reversed database, common contaminants, and proteins identified by a single modified peptide. Normalized protein intensities were log2 transformed. The proteins were filtered to retain those quantified in 3 biological replicates of control or TMG treated cells. Missing values were imputed from a normal distribution with a width of 0.3 and down shift of 1.8 from the total matrix. Log2 protein intensities from each condition were compared using a Student’s *t*-test. The log2 fold changes, *p* values, and q values are provided.

Identification of O-GlcNAc modified proteins. Protein extracts from thiamet G treated MDA-MB-231 cells were prepared as described above. Following trypsin digestion, N-linked glycans were removed with peptide-N-glycosidase F (PNGase F) PRIME (N-Zyme Scientifics, Doylestown, PA, USA), at 37 °C and 300 rpm for 18 h (1:100 enzyme:protein). The reaction was inactivated with formic acid (FA) to a final concentration of 1% followed by centrifugation at 16,000× *g* for 10 min to remove debris. Peptides were desalted with 500 mg sorbent Sep-Pak tC18 6 cc cartridges (Waters WAT036790) according to the manufacturer’s instructions and dried under vacuum. For high pH RP fractionation, peptides were resuspended in 2 mL of 50 mM ammonium bicarbonate and separated at 3 mL/min on a Zorbax 300 Å Extend-C18 column (9.4 × 250 mm, 5 µm, 300 Å, Agilent Technologies, Santa Clara, CA, USA) using a gradient of 1–25% B in 50 min, 25–60% in 4 min, 60–70% in 2 min, and 70% B for 9 min (Solvent A: 50 mM ammonium bicarbonate; Solvent B: 10% 50 mM ammonium bicarbonate with 90% acetonitrile (*v*/*v*)). One hundred and twenty 1.5 mL fractions were collected, brought to neutral pH with 10% FA, concatenated into 10 fractions, and dried. Peptides were desalted with Sep-Pak tC18 3 cc cartridges (Waters), dried by vacuum centrifugation, and stored at −80 °C. O-GlcNAc modified peptides were immunoaffinity enriched using PTMScan O-GlcNAc [GlcNAc-S/T] Motif antibody kit (Cell Signaling, cat #95220) [24]. Anti-O-GlcNAc antibody beads were washed and resuspended in 160 μL of cold immunoaffinity purification (IAP) buffer. Peptides (~1 mg/fraction) reconstituted at 1 mg/mL in IAP buffer were incubated with 40 uL beads for 2 h at 4 °C with end-to-end rotation. Samples were centrifuged and supernatants deposited into clean tubes for a sequential enrichment using fresh antibody beads. Beads from the two sequential immunoaffinity steps were washed and peptides eluted with 0.15% trifluoroacetic acid were combined. Peptides from each fraction were desalted twice using C18 StageTips (Thermo Scientific) as described with the PTMScan O-GlcNAc [GlcNAc-S/T] Motif Kit. Samples were dried by vacuum centrifugation and stored at −80 °C. LC-MS/MS was performed as above with the following changes in instrument parameters. MS2 spectra of ions with charge state 2–6 were acquired in data dependent mode with alternating HCD and ETD and a cycle time of 3 s. HCD spectra were collected at 15,000 MS2 resolution, AGC target of 1 × 10^5^ (200% normalized AGC target), maximum ion injection time of 105 ms, and 40% collision energy. Electron transfer dissociation (ETD) spectra were acquired at 15,000 MS2 resolution, AGC target of 4 × 10^5^ (800% normalized AGC target), and maximum ion injection time of 120 ms using charge-dependent reaction times. The raw files were searched using MaxQuant version 2.4.2.0 (Max Plank Institute) against a human protein database downloaded 02022024 from UniProt as well as a database of common cell culture contaminants. Variable modifications specified were: HexNAc modification or phosphorylation of Ser, Thr, Tyr [25], oxidation of Met, and protein N-terminal acetylation. Within Andromeda, a customized modification was defined accounting for the neutral loss of GlcNAc and the diagnostic HexNAc oxonium ions (OGlcNAc_NL_Std). Identified peptides were filtered to 1% FDR using a decoy database strategy. A mininum score of 40 and differential score of 6 were required for modified peptides. Reporter ion quantification was used to extract intensities of the diagnostic HexNAc ions (138, 144, 168, 186, 204) from the spectra. O-GlcNAc peptides with a measured oxonium ion intensity ratio characteristic of GlcNAc (138/144 > 3) rather than GalNAc are indicated [26,27]. Reporter ion #6 was included to extract intensities for *m*/*z* 366 (HexNAc-Hex) to remove peptides with a higher level of glycosylation. Putative O-GlcNAcylated peptides were filtered to retain those with at least 3 HexNAc oxonium ions including 204 *m*/*z* in HCD spectra. The probabilities of O-GlcNAc site assignment are reported.

### 2.9. Identification of O-GlcNAc Sites of GATAD2B

Immunoprecipitated, gel-purified GATAD2B was reduced with dithiothreitol, alkylated with iodoacetamide, and in-gel digested with trypsin. Extracted peptides were analyzed by LC-MS/MS as described above with the following modifications. HCD fragmentation was performed with a precursor isolation window of 1.7 *m*/*z*, 200% normalized AGC, a maximum injection time of 105 ms, and HCD collision energy of 40%. An HCD product ion of 204.0867 within the top 20 ions with a 15 ppm mass tolerance was used to trigger acquisition of an ETD scan. ETD fragmentation was performed using a 1.7 *m*/*z* isolation window, 15,000 resolution, 120 ms maximum injection time, 800% normalized AGC target, using the calibrated charge dependent ETD reaction parameters.

The raw files were searched using MaxQuant version 2.0.1.0 (Max Plank Institute) as described above against human GATAD2B downloaded from UniProt. All reported O-GlcNAc modified peptides of GATAD2B were manually verified. A previously identified site of O-GlcNAcylation on tyrosine within GATAD2B was not observed [25]. HCD MS/MS spectra contained the diagnostic GlcNAc fragmentation pattern with complete neutral loss of the monosaccharide from the peptide precluding site assignment. Peptide fragmentation by ETD was insufficient for site assignment.

### 2.10. Detection of GATAD2B Protein Interactions

MDA-MB-231 cells were cultured in Dulbecco’s Modified Eagle’s Medium (Corning 10-014-CV) supplemented with 10% fetal bovine serum. Cells were maintained in a humidified atmosphere of 5% CO_2_ at 37 °C and were passaged by trypsinization every two to three days. Cells at 90–95% confluency were treated with Thiamet G (Sigma-Aldrich) at a final concentration of 10 µM or OSMI-1 (Sigma-Aldrich) at a final concentration of 37 µM, or DMSO. After 24 h, cells were washed three times with cold PBS and lysed with cell lysis buffer (Cell Signaling #9803S) amended with universal nuclease (Thermo Scientific) and brought to a final concentration of 200 mM NaCl. Protein concentration was determined using BCA assay. One mg of protein was incubated with anti-GATAD2B antibody (Bethyl, Laboratories, Montgomery, TX, USA, cat#A301-281A) or rabbit IgG (Cell-Signaling, cat# 2729) as negative control overnight at 4 °C. Washed protein A/G Plus Agarose beads (Santa-Cruz, cat# sc2003) (30 uL) were added and protein complexed immunoprecipitated for 2 h in at 4 °C. Beads were washed wih 25 mM Tris pH 7.4, 150 mM NaCl, 1 mM EDTA, 1% NP40, 5% Glycerol) five times and once with PBS. Proteins were eluted with 3× SDS sample loading buffer (60 uL) and denatured at 95 °C for 8 min. Proteins were separated by 4–20% glycine SDS gel and each lane was in-gel trypsin digested, cysteines were reduced and alkylated with iodoacetamide. Extracted peptides were separated and analyzed on an EASY nLC 1200 System (Thermo Scientific) in-line with the Orbitrap Exploris 480 (Thermo Scientific) with instrument control software v. 4.2.28.14. Two µg of peptides were pressure loaded onto a C18 reversed phase column (Acclaim PepMap RSLC, 75 µm × 25 cm (2 µm, 100 Å) ThermoFisher cat. # 164941) and separated using a gradient of 0–35% B in 90 min (Solvent A: 5% acetonitrile, 0.2% formic acid; Solvent B: 80% acetonitrile, 0.2% formic acid) at a flow rate of 300 nL/min.

Data were acquired in data-dependent acquisition (DDA) and data independent acquisition (DIA) modes to build a hybrid DDA and DIA library for database searching. High-resolution massspectometry analysis was carried out using FTMS with a survey scan of 60,000, in a mass range of *m*/*z* 375–1575. The mass spectra of the most abundant precursors were collected every 3 s. For the survey MS scan, automatic gain was set at 300%. For the MS/MS scan, automatic gainwas set at 100%. HCD fragmentation was carried out with 1.4 *m*/*z* precursor isolation window, 40 ms maximum injection time and 33% HCD collision energy. MS/MS data was collected at 15,000 resolution with peptide-specific monoisotopic-precursor selection. Resequencing for 20 s was carried out and precursors within 10 ppm mass tolerance were dynamically excluded. Advanced peak determination was enabled, and precursor ions with charged state of 1, >6 or undetermined were excluded. Mass spectra were also acquired in data-independent mode with a high resolution (60,000) FTMS survey scan, mass range of *m*/*z* 380–985, with an automatic gain control target value of 100% and a maximum injection time of 100 ms. The AGC target value for fragment spectra was set at 200%. 59 windows of 10 *m*/*z* scanning from 380–980 was used. The resolution was 15,000 and injection time 40 ms. HCD collision energy of 33%.

Data were searched using Spectronaut 17 using the Direct DIA+ algorithm (Biognosys, Newton, MA, USA) against a reviewed human protein database downloaded from Uniprot on (20 June 2022 with 20,386 entries). A decoy database was generated in Spectronaut and a contaminant database (downloaded with MSFragger) was included. Two peptides were required for protein identification. Data were uploaded into Perseus v1.6.15.0 (Max Planck Institute) for data processing and annotation with complexes from the Corum database (4.1 release November 2022), Reactome pathways (downloaded 03172023), Go terms and Uniprot gene names (downloaded from Uniprot 080520_SP_HUMAN_n20353). Binary comparisons were performed between GATAD2B IP vs IgG control; GATAD2B_ThiametG IP vs IgG control; and GATAD2B_OSMI IP vs IgG control. For each binary comparison, log2 transformed protein intensities were filtered to retain proteins observed in all 3 replicates of the GATAD2B IP. Missing values for the IgG controls were imputed with 0.3 width and 1.8 downshift from the total matrix. A paired two sample *t*-test was performed against control samples run on the same gel. The log2 fold change versus the –log10 *p*-values were plotted in volcano plots.

### 2.11. Co-Immunoprecipitation

Cells were allowed to grow in monolayer and were treated with 100 µM of OGT inhibitor OSMi or with control of DMSO for 24 h. Cells were then harvested, washed and lysed in RIPA lysis buffer as described above. Bradford assay was used to quantify protein concentration. 1000 µg of protein was incubated with anti-GATAD2B (Bethyl, cat #A301-281A) and washed protein A/G Plus Agarose (Santa-Cruz, cat #sc2003) for overnight at 4 °C. Normal rabbit IgG (Cell-Signaling, cat #2729) was used as control. After incubation, samples were centrifuged and extensively washed with RIPA buffer before analyzing by Western blot as described above. Indicated targets were detected using the following specific antibodies: Anti-GATAD2B (Cell-Signaling, cat #73098), anti-ITCH (Cell-Signaling, cat #12117), anti-HA (Cell-Signaling, cat #3724), anti-Ubiquitin (Cell-Signaling, cat #3933).

To identify the Ubiquitination level of Wild-type and mutant GATAD2B, HEK-293T cells were transfected with plasmid encoding for GATAD2B-WT or GATAD2B-Mut. Cancer cells expressing GATAD2B-WT or GATAD2B-Mut were washed and lysed using RIPA buffer supplemented with protease inhibitors. 1000 µg from each sample were used for immunoprecipitation using anti-GATAD2B (Bethyl, cat #A301-281A) as described above. Immunoprecipitated proteins were resolved and analyzed by Western blot using the following indicated antibodies: Anti-GATAD2B (Cell-Signaling, cat #73098), anti-Ubiquitin (Cell-Signaling, cat #3933).

To confirm the role of ITCH in Ubiquitinating GATAD2B, breast cancer cells were infected with lentiviral vector delivering control or ITCH shRNA. Cells were then treated with OGT inhibitor OSMi (100 µM) for 24 h before being collected for cell lysate. 1000 µg from each sample were used for immunoprecipitation using anti-GATAD2B (Bethyl, cat #A301-281A) as described above. Immunoprecipitated proteins were analyzed by Western blot using the following indicated antibodies: Anti-GATAD2B (Cell-Signaling, cat #73098), anti-ITCH (Cell-Signaling, cat #12117), anti-Ubiquitin (Cell-Signaling, cat #3933).

### 2.12. Succinylated Wheat Germ Agglutinin Assay

Cells were allowed to grow in monolayer as described before and was treated with 2 µM of OGA inhibitor Thiamet-G (TMG) for 3 h to increase the level of total O-GlcNAc. After 3 h, cells were collected, and cell lysates were prepared as described above. 250 µg of protein from each sample was incubated with washed succinylated Wheat Germ Agglutinin Agarose (sWGA) beads (Vector Laboratories, cat #AL-1023S-5) for overnight at 4 °C. A sample with sWGA bead prebound to N-acetylglucosamine (GlcNAc) (Sigma, cat #A8625) was used as a negative control. After incubation, beads were pelleted and extensively washed before analyzing by Western blot as described above. The following specific antibodies were used to detect proteins of interest: Anti-GATAD2B (Cell-Signaling, cat #73098), anti-Sp1 (Cell-Signaling, cat #5931), anti-HA (Cell-Signaling, cat #3724).

### 2.13. Quantitative RT-PCR (qRT-PCR)

Breast cancer cells were collected for total RNA extraction using Trizol reagent (Invitrogen) as per manufacturer’s instruction. Briefly, Trizol reagent was added directly to monolayer of breast cancer cells. The mixture of cells and Trizol was transferred into a new tube, mixed with cloroform to extract total RNA. Isopropanol was added to the RNA fraction to precipitate RNA, which was subsequently washed with 70% ethanol. RNA pellet was allowed to air-dry and resuspended in nuclease-free water. RNA concentration was determined by measuring OD260 nm using NanoDrop. mRNA level of target genes was assessed by qRT-PCR using Brilliant II Master Mix Kit (Agilent) as per manufacturer’s instruction. qRT-PCR assay was conducted using qRT-PCR Applied Biosystem 7500 (Applied Biosystem, Waltham, MA, USA). The following Taqman probes were used to quantify mRNA level of targeted genes: PPIA (Hs04194521_s1), OCT4 (Hs04260367), SOX2 (Hs04234836_s1), c-MYC (Hs00905030_m1), Nanog (Hs02387400_g1) and GATAD2B (Hs00372672_m1).

### 2.14. Apoptosis Assay

Breast cancer cells growing in monolayer were treated with increasing concentration of paclitaxel or with control of DMSO for 48 h. All cells were harvested and washed with PBS. Cells were analyzed using Annexin V-FITC Apoptosis detection kit (Biolegend, San Diego, CA, USA) as per manufacturer’s instruction. In brief, 10^6^ cells were resuspended in 1 mL of 1× provided binding buffer to final concentration of 10^6^ cells/mL. 100 μL of cell suspension was mixed with 5 μL of provided FITC-Annexin V and 5 μL of Propidium Iodide and incubated for 15 min in the dark at room temperature. After incubation, the volume of each sample was brought up to 500 μL using 1× binding buffer. Flow cytometry analysis was conducted using Guava flowcytometry system (MillporeSigma). Unstained and single stain samples were used to determine the basal fluorescence signal.

### 2.15. Clonogenic Survival Assay

Breast cancer cells growing in monolayer were treated with increasing dose of paclitaxel, or with control DMSO for 48 h. Cells were harvested and 1000 cells from each sample were plated and allowed to grow for 10–14 days to form colonies. Colonies were subsequently stained with crystal violet and counted. Counted number of colonies was normalized against control of DMSO treatment.

### 2.16. Database Analysis

OncoPrint plot of genes coding for NuRD complex was generated using available online database analysis platform cBioportal (https://www.cbioportal.org accessed on 31 March 2023) using the Breast cancer METABRIC database (Invasive Breast Carcinoma: METABRIC, Nature 2012 and Nat Commun 2016). OncoPrints of GATAD2B, GATAD2A, CHD4, MTA2, HDAC2 and RBBP4 were analyzed. Correlation in expression of GATAD2B and OGT was analyzed using cBioportal platform, using the breast cancer METABRIC database. Co-expression of OGT mRNA was plotted against GATAD2B mRNA [28,29].

GATAD2B level in breast cancer subtypes was analyzed using available online database at UALCAN (https://ualcan.path.uab.edu/analysis.html, accessed on 31 March 2023) using CPTAC database (Breast cancer, total-protein, major subclasses) [30].

Kaplan-Meier survival plots were created using Kaplan-Meier plotter, an available online database analysis platform (https://kmplot.com/analysis, accessed on 31 March 20233) [31]. The relevant mRNA level of GATAD2B in basal-type breast cancer (auto select best cutoff, overall survival, PAM50 subtype: basal) was plotted against the overall survival of breast cancer patients. The relevant protein level of GATAD2B in all breast cancer (auto select best cutoff, overall survival, Tang_2018) was plotted against overall survival of breast cancer patients. The expression of GATAD2B and relevant ROC plot in breast cancer patients, which differentially responded to any chemotherapy in five years (relapse-free survival at 5 years, any chemotherapy), were generated using available online ROC plotter (https://www.rocplot.org/site/treatment, accessed on 31 March 2023) [32].

### 2.17. Statistical Analysis

Data from at least 3 biological repeats is shown as mean ± SEM. Statistical analysis was conducted using Graphpad Prism 9.0 and representative *p*-value was presented as * *p* < 0.05, ** *p* < 0.01, *** *p* < 0.001.

## 3. Results

### 3.1. Hyper-O-GlcNAcylation Increases Expression of NuRD Complex Protein GATAD2B in Breast Cancer Cells

**Figure 1:**
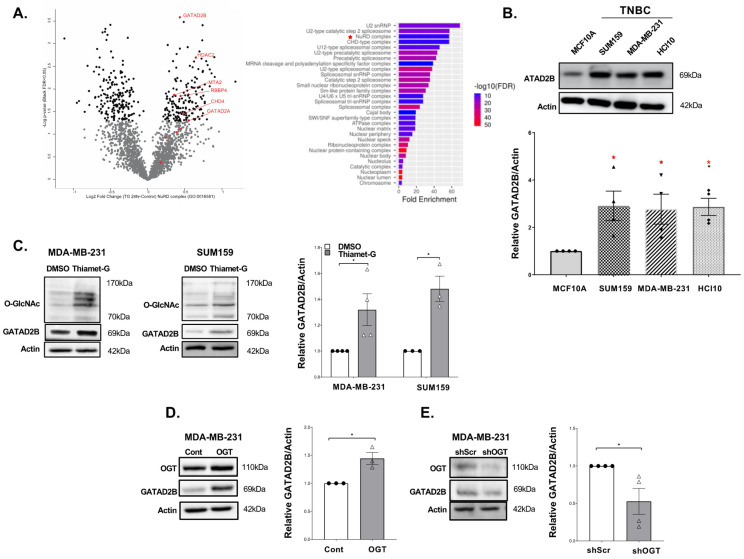
NuRD complex protein GATAD2B is regulated by OGT/O-GlcNAc in breast cancer cells. (**A**)-Mass spectrometric analysis of TNBC cells MDA-MB-231, treated with DMSO or OGA inhibitor Thiamet-G for 24 h, shows changes in protein level with 157 proteins down-regulated and 142 protein up-regulated (left). GO-analysis of up-regulated proteins (ShinyGO 0.75: GO-cellular components) shows enrichment of NuRD complex in Thiamet-G treatment (right). (**B**)-Immunoblot analysis of different TNBC cell lines shows elevated level of GATAD2B in TNBC cells compared to control of immortalized mammary gland cells MCF-10A (top and quantified graph shows significantly higher level of GATAD2B in TNBC compared to control of MCF-10A cells (bottom). One sample *t*-test against hypothetical value is reported as mean ± SEM, * *p* < 0.05. (**C**)-Immunoblots of TNBC cells MDA-MB-231 and SUM159, treated with DMSO or OGA inhibitor Thiamet-G for 24 h, show elevated level of GATAD2B in Thiamet-G treatment compared to control of DMSO (left and middle), and quantified graph showing significant increase in GATAD2B level in the presence of Thiamet-G compared to DMSO (right). One sample *t*-test against hypothetical value is reported as mean ± SEM, * *p* < 0.05. (**D**)-Immunoblots of TNBC MDA-MB-231 cells, control or with OGT overexpression, show increase in GATAD2B level in OGT overexpression, compared to control (left), and quantified graph shows significant increase in GATAD2B level in OGT overexpression, compared to control (right). One sample *t*-test against hypothetical value is reported as mean ± SEM, * *p* < 0.05. (**E**)-Immunoblots of TNBC MDA-MB-231 cells, transduced with control or OGT shRNA, show decrease in GATAD2B level in OGT knockdown, compared to control (left), and quantified graph shows significant decrease in GATAD2B level in OGT knockdown, compared to control (right). One sample *t*-test against hypothetical value is reported as mean ± SEM, * *p* < 0.05.

Cancer stem cells contain hyper-O-GlcNAcylated proteins [17,33,34]. To identify proteins regulated by OGT and O-GlcNAc cycling that may contribute to CSC phenotypes, we treated MDA-MB-231 breast cancer cells with an OGA inhibitor Thiamet-G (TMG) to block the removal of the modification and increase the extent of protein O-GlcNAcylation. Changes in protein abundance were measured by mass spectrometry-based label free proteomics before and after 24 h TMG treatment. Differentially abundant proteins were determined using a Student’s *t*-test. Proteins with a *p* value < 0.05 were analyzed for enrichment of GO terms (ShinyGO v0.75) [35] (Figure 1A).

One highly enriched protein complex increasing in abundance with hyper-O-GlcNAcylation was the nucleosome remodeling and deacetylase (NuRD) complex. Changes in abundance of GATAD2B, GATAD2A, CHD4, MTA2, RBBP4 and HDAC2 reached a *p* value < 0.05, with GATAD2B and HDAC2 reaching an adjusted *p* value, FDR < 0.05 (Supplementary Table S1). We confirmed these results by immunoblot analysis and showed a significant increase in the level of NuRD complex proteins in TMG treated breast cancer cells compared to controls with the exception of RBBP4 (Supplementary Figure S1A). Direct LC-MS/MS analysis of the immunoaffinity enriched O-GlcNAcome from TMG treated cells revealed 813 putative O-GlcNAc modified peptides including a novel O-GlcNAcylated peptide of GATAD2B, the only member of the NURD complex observed (Supplementary Table S2). Database analysis (cBioportal, breast cancer: METABRIC) showed some alterations in breast cancer samples for these NuRD complex components. However, GATAD2B stood out as it was amplified or overexpressed in nearly 20% of human breast cancer samples (Supplementary Figure S1B). High expression of GATAD2B was observed at the protein level in both luminal and triple negative breast cancer (TNBC) patients (Supplementary Figure S1C). Consistent with these results, we found a significant increase in GATAD2B protein levels in TNBC cell lines compared to non-transformed mammary epithelial MCF10A cells (Figure 1B). Importantly, breast cancer patients containing high expression of GATAD2B RNA (Supplementary Figure S1D) and protein (Supplementary Figure S1E) were associated with poor survival rates. Our analysis of other NURD proteins that were also elevated in Figure 1A, including CHD4, GATAD2A, HDAC2. MTA2 and RBBP4 did not show altered expression in breast cancer patient databases thus we focus this study on the role of GATAD2B on breast cancer cells. Increased O-GlcNAc levels significantly elevated the level of GATAD2B in MDA-MB-231 and SUM159 cells treated with TMG (Figure 1C). Consistent with this data, overexpression of OGT significantly increased GATAD2B protein levels in MDA-MB-231 (Figure 1D) and SUM159 (Supplementary Figure S1F) cells compared to control cells. Conversely, targeting OGT genetically via RNAi significantly reduced GATAD2B levels in MDA-MB-231 (Figure 1E) and SUM159 (Supplementary Figure S1G) cells compared to control cells. Thus, GATAD2B levels are increased in aggressive breast cancer cells, can be regulated by O-GlcNAcylation and associate with poor survival in human breast cancer patients.

### 3.2. GATAD2B Regulates Cancer Stem-like Cells Phenotypes

**Figure 2:**
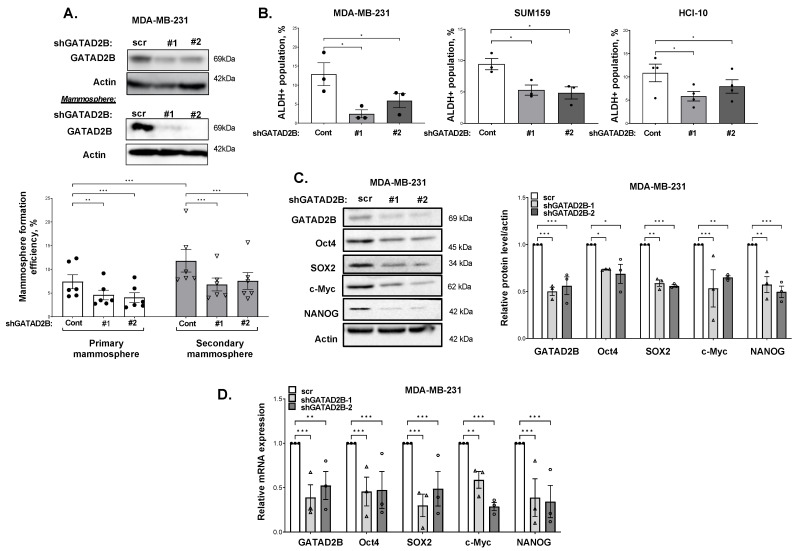
GATAD2B is critical to maintain CSCs phenotype of breast cancer cells. (**A**)-TNBC MDA-MB-231 cells were transduced with control (scramble) or with GATAD2B-specific shRNAs. Lysates from control or transduced with GATAD2B shRNAs MDA-MB-231 cells growing in monolayer (top) or in mammosphere (bottom) were collected for immunoblot analysis using indicated antibodies. Control or transduced with GATAD2B shRNAs MDA-MB-231 cells were allowed to grow in mammosphere formation assay. Mammospheres were counted and primary mammosphere formation efficiency from each condition was determined and graphed (bottom). Primary mammospheres were collected and regrown in the same mammosphere culture condition to form secondary mammosphere. The number of mammospheres and secondary mammosphere formation efficiency from each condition was determined and graph (right). Two-way ANOVA with Sidak test is reported as mean ± SEM, ** *p* < 0.01, *** *p* < 0.001. (**B**)-Quantified graph of ALDH+ CSCs population from TNBC cells MDA-MB-231, SUM159 and PDX cells HCI-10, which were transduced with scramble or GATAD2B shRNAs. One-way ANOVA with Sidak test is reported as mean ± SEM, * *p* < 0.05. (**C**)-Lysates from MDA-MB-231 cells transduced with scramble or GATAD2B shRNAs were collected for immunoblot analysis using indicated antibodies (left). Quantified graph of blots from MDA-MB-231 cells with control or GATAD2B shRNAs (right). Multiple one sample *t*-test against hypothetical value with Holm-Sidak correction is reported as mean ± SEM, * *p* < 0.05, ** *p* < 0.01, *** *p* < 0.001. (**D**)-Total RNA of MDA-MB-231 cells transduced with scramble or GATAD2B shRNAs were collected and mRNA level of indicated genes were analyzed by qRT-PCR. The level of PPIA was used as internal control. Multiple one sample *t*-test against hypothetical value with Holm-Sidak correction is reported as mean ± SEM, ** *p* < 0.01, *** *p* < 0.001.

Given the role of NuRD proteins in regulating self-renewal and differentiating activity of stem cells [36], we hypothesized that GATAD2B may contribute to CSC phenotypes. To evaluate the potential role of GATAD2B in regulating cancer stem-like cells in breast cancer cells, protein level of GATAD2B in cancer stem-like cells enriched mammospheres was evaluated. Mammosphere assays enrich for cancer stem-like cells as these cells possess the self-renewal capacity required to sustain growth under non-adherent and serum free conditions [37]. The level of GATAD2B was significantly higher in mammosphere cultured breast cancer cells including MDA-MB-231, SUM159 and a patient-derived-xenograft (PDX) cell lines HCI-10, compared to control cells grown in adherence under similar media conditions (Supplementary Figure S2A), suggesting a potential role of GATAD2B in regulating cancer stem-like cells properties. To evaluate this further, expression of GATAD2B in breast cancer cells was inhibited by shRNA and stem-like cells properties of cancer cells were assessed by primary and secondary mammosphere formation assay. The ability of cells to generate secondary mammospheres upon repeated passaging is a hallmark of stemness, as it demonstrates sustained self-renewal [38]. Reducing GATAD2B expression with stable expression of RNAi significantly reduced both primary and secondary mammosphere formation in MDA-MB-231 cells compared to control cells (Figure 2A). Similar results were also observed in breast cancer cells SUM159 (Supplementary Figure S2B) and HCI-10 (Supplementary Figure S2C), suggesting GATAD2B may play a role in regulating stemness. Since the number of mammospheres reflects the abundance of cancer stem-like cells [39], we tested whether reducing GATAD2B levels alters the cancer stem-like cell population.

To test that idea, breast cancer cells were transduced with GATAD2B shRNA or control shRNA, and the population cancer stem-like cells was assessed by ALDEFLOUR staining [40]. Reduced expression of GATAD2B in MDA-MB-231, SUM159, and HCI-10 cell lines resulted in a significant decrease in the population of ALDH+ cancer stem-like cells compared to control cells (Figure 2B). Reduction in cancer stem-like cell population was further confirmed by using a SORE-GFP reporter where SOX2 and Oct4 response elements drive GFP expression previously shown to associate with elevated self-renewal and tumor initiation capacities [23]. Similar to the ALDEFLOUR staining, knockdown of GATAD2B significantly reduced the population of SORE-GFP+ in MDA-MB-231 cells compared to controls (Supplementary Figure S2D). Together, these data indicate that GATAD2B plays a critical role in maintaining the cancer stem-like cells properties in multiple breast cancer cell lines.

Cancer stem-like cells phenotype is regulated by a set of CSC-associated transcription factors including c-Myc, OCT4, SOX2 and NANOG which are known to drive cancer progression [41,42]. To understand whether GATAD2B was regulating key CSC-associated factors, we probed for changes in these factors. In MDA-MB-231 cells, GATAD2B knockdown significantly reduced expression of cancer stem-like cells factors OCT4, SOX2, c-Myc and NANOG, at both protein (Figure 2C) and mRNA level (Figure 2D), further implicating the role of GATAD2B in maintaining cancer stem-like cells phenotype in breast cancer cells. Importantly, genetically inhibition of GATAD2A, an analog of GATAD2B in the NuRD complex, did not alter mammosphere formation in MDA-MB-231 cells (Supplementary Figure S2E). Altogether, this data indicates that GATAD2B, but not GATAD2A, regulates cancer stem-like cell populations and cancer stem cell factors in breast cancer cells.

**Figure 3:**
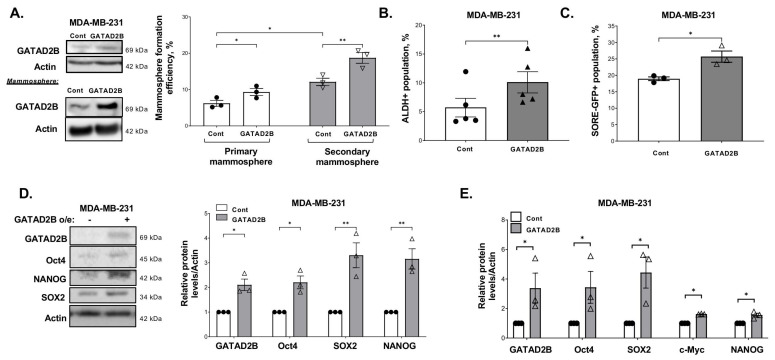
GATAD2B promotes CSCs phenotype of breast cancer cells. (**A**)-Lysates from control or GATAD2B overexpressing MDA-MB-231 cells growing in monolayer (top) or in mammosphere (right) were collected for immunoblot analysis using indicated antibodies. Control or GATAD2B overexpressing MDA-MB-231 cells were allowed to grow in mammosphere formation assay. Mammospheres were counted and primary mammosphere formation efficiency from each condition was determined and graphed (right). Primary mammospheres were collected and regrown in the same mammosphere culture condition to form secondary mammosphere. The number of mammospheres and secondary mammosphere formation efficiency from each condition was determined and graph (right). Two-way ANOVA with Sidak test is reported as mean ± SEM, * *p* < 0.05, ** *p* < 0.01. (**B**)-Quantified graph of ALDH+ CSCs population from control or GATAD2B overexpressing TNBC cells MDA-MB-231. Paired *t*-test is reported as mean ± SEM, ** *p* < 0.01. (**C**)-Quantified graph of SORE-GFP+ CSCs population from control or GATAD2B overexpressing TNBC cells MDA-MB-231. Paired *t*-test is reported as mean ± SEM, * *p* < 0.05. (**D**)-Lysates from control or GATAD2B overexpressing MDA-MB-231 cells were collected for immunoblot analysis using indicated antibodies (left). Quantified graph of blots from control or GATAD2B overexpressing MDA-MB-231 cells (right). Multiple one sample *t*-test against hypothetical value with Holm-Sidak correction is reported as mean ± SEM, ** *p* < 0.01, * *p* < 0.05. (**E**)-Total RNA of control or GATAD2B overexpressing MDA-MB-231 cells were collected and mRNA level of indicated genes were analyzed by qRT-PCR. The level of PPIA was used as internal control. Multiple one sample *t*-test against hypothetical value with Holm-Sidak correction is reported as mean ± SEM, * *p* < 0.05.

Since GATAD2B depletion attenuated CSC potential, we examined whether GATAD2B was sufficient to promote cancer stem-like cells phenotypes. We stably overexpressed GATAD2B in breast cancer cells and assessed the cancer stem-like cells properties. Overexpression of GATAD2B significantly increased both primary and secondary mammosphere formation in MDA-MB-231 cells (Figure 3A). Similarly, overexpression of GATAD2B also significantly increased mammosphere formation in SUM159 cells (Supplementary Figure S3A) and HCI-10 cells (Supplementary Figure S3B). Additionally, overexpression of GATAD2B also significantly increased the population of cancer stem-like cells detected by ALDEFLOUR staining in MDA-MB-231 cells (Figure 3B) and SUM159 cells (Supplementary Figure S3C) or by SORE-GFP reporter assay (Figure 3C) compared to controls. Overexpression of GATAD2B also significantly increased the expression of cancer stem-like markers at both protein (Figure 3D) and mRNA level (Figure 3E). Together, these data suggest a critical role of GATAD2B in regulating breast cancer stem-like cells.

### 3.3. GATAD2B Functions Downstream of OGT in Regulating Cancer Stem-like Cell Function

**Figure 4:**
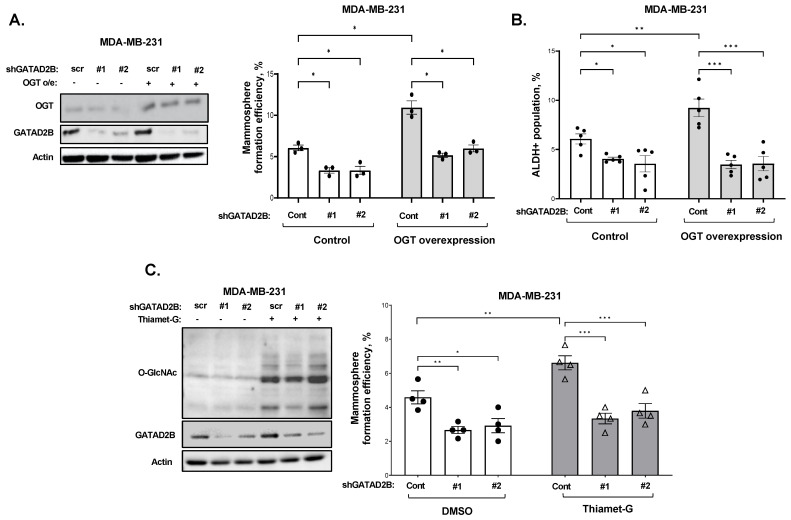
GATAD2B function downstream of OGT/O-GlcNAc in regulating CSCs in breast cancer. (**A**)-Lysates from control or OGT overexpressing MDA-MB-231 cells, transduced with scramble or GATAD2B shRNAs, were collected and analyzed by immunoblot using indicated antibodies (left). Control or OGT overexpressing MDA-MB-231 cells, transduced with scramble or GATAD2B shRNAs, were grown in mammosphere formation assay. The mammosphere formation efficiency in each condition was determined and presented in the quantified graph (right). Two-way ANOVA with Sidak test is reported as mean ± SEM, * *p* < 0.05. (**B**)-Quantified graph showing ALDH+ CSCs from control or OGT overexpressing MDA-MB-231 cells, transduced with scramble or GATAD2B shRNA. Two-way ANOVA with Sidak test is reported as mean ± SEM, * *p* < 0.05, ** *p* < 0.01. *** *p* < 0.001. (**C**)-Lysates from control or transduced with GATAD2B shRNAs MDA-MB-231 cells, treated with DMSO or OGA inhibitor Thiamet-G (2 µM) for 48 h, were collected and analyzed by immunoblot using indicated antibodies (left). Control or transduced with GATAD2B shRNAs MDA-MB-231 cells, treated with DMSO or OGA inhibitor Thiamet-G (2 µM) for 48 h, were grown in mammosphere formation assay. The mammosphere formation efficiency in each condition was determined and presented in the quantified graph (right). Two-way ANOVA with Sidak test is reported as mean ± SEM, * *p* < 0.05, ** *p* < 0.01. *** *p* < 0.001.

To test the potential connection between OGT and GATAD2B in regulating CSC function, we tested whether GATAD2B is required for OGT-mediated mammosphere formation. Consistent with previous studies [17], overexpression of OGT significantly increased mammosphere formation in breast cancer cells compared to control (Figure 4A).

However, reducing GATAD2B levels in breast cancer cells significantly reduced the OGT-mediated increase in mammosphere formation in MDA-MB-231 (Figure 4A) and SUM159 cells (Supplementary Figure S3D). We detected a similar inhibition in ALDEFLOUR staining in OGT overexpressing cells with stable reduction of GATAD2B compared to controls (Figure 4B). Consistent with this data, treatment of MDA-MB-231 cells with OGA inhibitor TMG mediated increase in mammosphere formation was significantly reduced in cells with stable GATAD2B knockdown (Figure 4C). Similar results were seen in SUM159 cells (Supplementary Figure S3D). Together, these data suggested that CSC regulation by OGT and O-GlcNAc requires, in part, GATAD2B expression.

### 3.4. O-GlcNAc Protects GATAD2B from ITCH-Mediated Proteasome-Dependent Degradation

**Figure 5:**
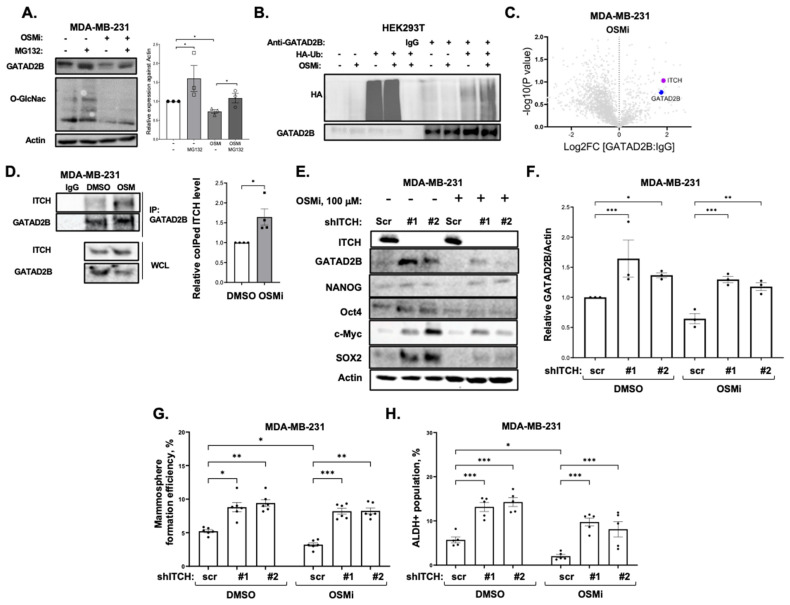
O-GlcNAc protects GATAD2B from ITCH-mediated proteasomal degradation. (**A**)-TNBC cells MDA-MB-231 were treated with DMSO or OGT inhibitor OSMi-1 (100 µM) for 24 h, then subsequently treated with DMSO or proteasome inhibitor MG-132 (10 µM) for an additional 24 h before collecting for immunoblot using indicated antibodies (left). Quantified graph of blots from MDA-MB-231 cells treated with DMSO, OSMi-1 or MG-132 (right). Two-way ANOVA with Sidak test is reported as mean ± SEM, * *p* < 0.05. (**B**)-HEK-293T cells, transfected with HA-tagged Ubiquitin, were treated with DMSO or with OGT inhibitor OSMi-1 (100 µM) for 24 h prior to lysate collection. Lysate from each sample was immunoprecipitated using anti-GATAD2B antibody or IgG and was subsequently analyzed by immunoblot using indicated antibodies. (**C**)-Volcano plot of mass spectrometry analysis showing ITCH being co-IPed together with GATAD2B in TNBC cells MDA-MB-231 by anti-GATAD2B antibody. (**D**)-TNBC cells MDA-MB-231 were treated with DMSO or OGT inhibitor OSMi-1 (100 µM) for 24 h, then were collected for immunoprecipitation using anti-GATAD2B antibody or IgG. Immunoprecipitated proteins were analyzed by Western blot using indicated antibodies (left). Quantified graph shows increase ITCH level being co-immunoprecipitated with GATAD2B in the presence of OGT inhibitor OSMi-1 (100 µM) compared to control of DMSO (right). One sample *t*-test against hypothetical value is reported as mean ± SEM, * *p* < 0.05. (**E**)-TNBC cells MDA-MB-231, transduced with scramble or ITCH shRNAs, were treated with DMSO or OGT inhibitor OSMi-1 (100 µM) for 48 h prior to being collected for immunoblot analysis using indicated antibodies. (**F**)-Quantified graph of (**E**) shows increase in the level of GATAD2B in the presence of ITCH shRNAs, compared to scramble shRNA, in TNBC cells MDA-MB-231 treated with DMSO or OGT inhibitor OSMi-1. Two-way ANOVA with Sidak test is reported as mean ± SEM, * *p* < 0.05, ** *p* < 0.01. *** *p* < 0.001. (**G**)-TNBC cells MDA-MB-231, transduced with scramble or ITCH shRNAs, were treated with DMSO or OGT inhibitor OSMi-1 (100 µM) for 48 h prior to being plated in mammosphere condition. Mammosphere formation efficiency from each condition was determined and graphed. Two-way ANOVA with Sidak test is reported as mean ± SEM, * *p* < 0.05, ** *p* < 0.01. *** *p* < 0.001. (**H**)-Quantified graph showing ALDH+ CSC from TNBC cells MDA-MB-231, transduced with scramble or ITCH shRNA and treated with DMSO or OGT inhibitor OSMi (100 µM). Two-way ANOVA with Sidak test is reported as mean ± SEM, * *p* < 0.05, *** *p* < 0.001.

Since GATAD2B plays a key role downstream of OGT, we further examined GATAD2B expression in the context of altered O-GlcNAcylation. Treatment with OGA inhibitor Thiamet-G significantly increased GATAD2B protein levels in MDA-MB-231 and SUM159 cells (Figure 1C) but did not change GATAD2B RNA levels as measured by quantitative RT-PCR (qRT-PCR) in these cells (Supplementary Figure S4A). To test whether O-GlcNAc regulates GATAD2B protein stability, breast cancer cells were treated with OGT inhibitor OSMi together with proteasome inhibitor MG-132. Treatment with OSMi significantly reduced the protein level of GATAD2B in MDA-MB-231 cells (Figure 5A).

However, that reduction in GATAD2B levels was partially rescued by MG-132 treatment, suggesting that OSMi-mediated inhibition of GATAD2B protein levels could be reversed by treatment of MG-132 (Figure 5A), suggesting that O-GlcNAc regulation of GATAD2B was proteasome dependent. Consistent with the idea that O-GlcNAc regulates GATAD2B via proteasomal pathway, we detected a 2.5-fold increase in GATAD2B ubiquitination under conditions of reduced O-GlcNAcylation in HEK293T cells (Figure 5B & Supplementary Figure S4B).

To identify a E3-ligase that may regulate GATAD2B ubiquitination, we immunoprecipitated GATAD2B from MDA-MB-231 cells treated with OGT inhibitor OSMi, OGA inhibitor Thiamet-G or with DMSO to enrich for proteins interacting with GATAD2B and analyzed by mass spectrometry. Label free proteomic results showed an increase in the level of ITCH, an E3-ligase, co-immunoprecipitated in OMSi treatment (Figure 5C, Supplementary Table S3) when compared to DMSO or TMG treatment (Supplementary Figure S4C), suggesting that ITCH may ubiquitinate GATAD2B in breast cancer cells. This interaction was confirmed by increased level of ITCH co-immunoprecipitated with GATAD2B in MDA-MB-231 cells treated with OSMi, compared to control treated with DMSO (Figure 5D). Consistent with idea that ITCH may serve as proteasomal regulator of GATAD2B, stable knockdown of ITCH with RNAi significantly increased GATAD2B protein levels in MDA-MB-231 cells compared to controls (Figure 5E,F). We tested whether ITCH may alter GATAD2B ubiquitination. ITCH knockdown significantly reduced GATAD2B ubiquitination in MDA-MB-231 cells compared to controls (Supplementary Figure S4D). ITCH knockdown also partly rescued OSMi treated reduction of GATAD2B protein levels (Figure 5E,F), further validated that ITCH regulates GATAD2B in an O-GlcNAc dependent manner.

Since targeting ITCH led to an increase in GATAD2B levels, we tested whether reducing ITCH levels could also increase CSC phenotypes. MDA-MB-231 cells containing stable knockdown of ITCH significantly increased mammosphere formation (Figure 5G), ALDEFLOUR staining (Figure 5H) and increased CSC factors SOX2, and c-Myc (Figure 5E) compared to control cells. Similar effects were seen in HCI-10 (Supplementary Figure S4E) and SUM159 (Supplementary Figure S4F) cells. We also tested whether targeting ITCH could reverse OGT inhibition-mediated effects on CSCs phenotypes. OSMi-mediated inhibition of cancer stem cell phenotypes including mammosphere formation (Figure 5G), ALDEFLOUR staining (Figure 5H) and expression of CSC factors c-Myc and SOX2 were partly reversed in cells containing ITCH knockdown compared to controls. Together, these data suggests that OGT and O-GlcNAc protects GATAD2B from proteasome-dependent degradation mediated by the E3 ligase ITCH and also identifies ITCH as a regulator of breast cancer stem cell functions.

### 3.5. GATAD2B O-GlcNAcylation Regulates Protein Stability and Cancer Stem-like Cell Phenotypes

**Figure 6:**
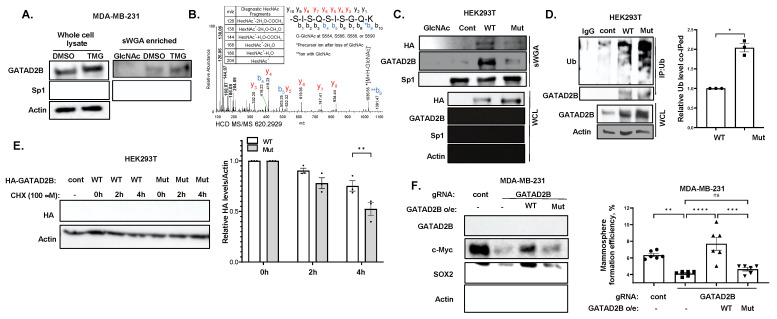
GATAD2B O-GlcNAcylation at the C-terminus is critical for protein stability and CSC function. (**A**)-TNBC cells MDA-MB-231 were treated with DMSO or OGA inhibitor Thiamet-G (2 µM) for 3 h prior to being collected for lysate. 250 µg of total protein from each condition was incubated with washed sWGA agarose bead overnight. The bead was then washed and analyzed by Western blot using indicated antibodies. For negative control we washed beads with incubated beads with GlcNAc to remove any O-GlcNAcylated proteins (lane 1). Sp1 was used as positive control. (**B**)-HEK-293T overexpressing GATAD2B was collected for immunoprecipitation using anti-GATAD2B antibody. Immunoprecipitated GATAD2B was subsequently analyzed using mass spectrometry. Mass spectrometry result shows detected O-GlcNAc modification at the C-terminus of GATAD2B. (**C**)-HEK-293T cells were transfected with HA-tagged wild-type (WT) GATAD2B or GATAD2B with S584/586/588/590A mutations (Mut) and were subsequently collected for lysate. Lysate from HEK-293T cells, control plasmid or overexpression WT or Mutant GATAD2B, were incubated with sWGA agarose bead overnight before analyzing by Western blot using indicated antibodies. For negative control we washed beads with incubated beads with GlcNAc to remove any O-GlcNAcylated proteins (lane 1). (**D**)-HEK-293T cells were transfected with WT or Mutant GATAD2B and were subsequently collected for immunoprecipitation using anti-GATAD2B antibodies. Immunoprecipitated proteins were analyzed by Western blot using indicated antibodies (left). Quantified graph shows increase in the level of poly-Ubiquitination co-immunoprecipitated with Mutant GATAD2B, compared to WT GATAD2B (right). One sample *t*-test against hypothetical value is reported as mean ± SEM, * *p* < 0.05. (**E**)-HEK-293T cell were transfected with control plasmid (lane 1), HA-tagged WT or Mutant GATAD2B and were subsequently treated with Cycloheximide (100 µM) for 0, 2 and 4 h before being collected for Western blot using indicated antibodies (left). Quantified graph shows decrease in the level of HA-tagged Mutant GATAD2B compared to WT GATAD2B overtime (right). Two-way ANOVA with Sidak test is reported as mean ± SEM, ** *p* < 0.01. (**F**)-TNBC cells transduced with control or GATAD2B-specific gRNA were infected with lentiviral vector containing WT or Mutant GATAD2B and were subsequently collected for Western blot using indicated antibodies (left). Quantified graph shows mammosphere formation efficiency of control or transduced with GATAD2B gRNA MDA-MB-231 cells overexpressing WT or Mutant GATAD2B (right). Two-way ANOVA with Sidak test is reported as mean ± SEM, ns—not significant, ** *p* < 0.01, *** *p* < 0.001, **** *p* < 0.001.

To further understand the mechanism of GATAD2B regulation by O-GlcNAcylation, we confirmed O-GlcNAcylation of GATAD2B was dynamically modulated by O-GlcNAc cycling enzymes in breast cancer cells. MDA-MB-231 cells were treated with DMSO or with TMG followed by O-GlcNAc enrichment by Succinylated Wheat Germ Agglutinin (sWGA). Enriched O-GlcNAc proteins were then resolved and analyzed by immunoblot for GATAD2B. GATAD2B was detected among those enriched by sWGA and the intensity of enriched GATAD2B was elevated by TMG treatment, verifying that GATAD2B could be directly modified with O-GlcNAc in breast cancer cells (Figure 6A). Mass spectrometric analysis of immunoprecipitated GATAD2B showed O-GlcNAc modification of peptides 521–527 GIPTSAR and 584–593 SISQSISGQK (Figure 6B, Supplementary Table S4) in addition to 250–264 SATNTTLPHMLMSQR observed by O-GlcNAcomics Given that O-GlcNAc modification of peptide 584–593 has been shown to increase with OGA inhibition in embryonic stem cells, brain tissue, and Hela cells [43], we examined the impact of site-directed mutagenesis within this C-terminal peptide of GATAD2B.

To evaluate the functional role of GATAD2B O-GlcNAc modification in regulating cancer stem-like cells, all four modified Serine residues (584, 586, 588, and 590) were mutated to Alanine. The mutant (Mut) GATAD2B showed reduced O-GlcNAcylation in sWGA enrichment assay, when compared to wild-type (WT) GATAD2B (Figure 6C). To test whether GATAD2B O-GlcNAcylation altered its ubiquitination, we compared ubiquitination levels between overexpressed GATAD2B-WT and GATAD2B-Mut and found a two-fold increase in ubiquitination in GATAD2B-Mut compared to GATAD2B-WT (Figure 6D) suggesting that O-GlcNAc modification of GATAD2B C-terminus protects the protein from ubiquitination. Consistent with this data, the half-life of mutant GATAD2B was reduced compared to GATAD2B-WT in cells under cycloheximide treatment (Figure 6E). Thus, GATAD2B O-GlcNAcylation protects from ubiquitination and degradation.

To evaluate the role of GATAD2B O-GlcNAcylation on regulating cancer stem-like cells phenotypes, MDA-MB-231 cells were infected with GATAD2B gRNA to inhibit expression of endogenous GATAD2B. WT or Mut GATAD2B were stably overexpressed in cells and cancer stem-like cells properties were analyzed. Consistent with RNAi results, infection with GATAD2B gRNA reduced GATAD2B levels and levels of cancer stem-like cells markers SOX2 and c-Myc, and significantly reduced mammosphere formation when compared to control gRNA (Figure 6F). Importantly, re-expression of WT GATAD2B, but not GATAD2B-Mut, rescued expression of cancer stem-like cells markers, as well as mammosphere formation in MDA-MB-231 (Figure 6F) and HCI-10 cells (Supplementary Figure S5A,B). These data suggested that GATAD2B O-GlcNAcylation plays a critical role in promoting cancer stem-like cell properties in breast cancer cells.

### 3.6. GATAD2B Expression Regulates Chemoresistance in Breast Cancer Cells

**Figure 7:**
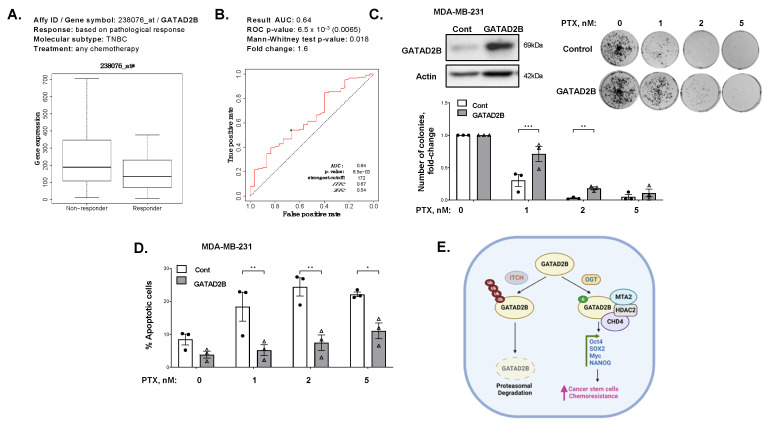
GATAD2B promotes paclitaxel resistance in TNBC cells. (**A**)-Plot showing expression in breast cancer patients responded differentially against. (**B**)-ROC-plot of GATAD2B expression in breast cancer patients, responded differentially against chemotherapy. (**C**)-TNBC MDA-MB-231 cells, control or overexpressing GATAD2B, were harvested for Western blot using indicated antibodies (left). MDA-MB-231 control or GATAD2B overexpressing cells were treated with an increasing dose of paclitaxel, or with control DMSO for 48 h. Cells were plated in clonogenic assay and were allowed to grow for 10–14 days. Colonies were stained, counted. Representative images show stained colonies (right). Quantified graph shows increased number of colonies in GATAD2B overexpression compared to control cells (bottom). Two-way ANOVA with Sidak test is reported as mean ± SEM, ** *p* < 0.01. *** *p* < 0.001. (**D**)-Quantified graph showing percentage of dead cells from control or GATAD2B overexpressing MDA-MB-231 cells treated with increasing dose of paclitaxel for 48 h. Two-way ANOVA with Sidak test is reported as mean ± SEM, * *p* < 0.05, ** *p* < 0.01. (**E**)-Schematic model of GATAD2B regulation by OGT and ITCH in breast cancer. ITCH mediates ubiquitination of GATAD2B and its degradation. OGT directly modifies GATAD2B protecting it from being ubiquitinated by ITCH and from degradation, thus promoting CSC phenotype and chemoresistance in breast cancer.

One notable characteristic of cancer stem-like cells is the ability to resist cell death caused by chemotherapeutic drugs [44]. To evaluate the potential role of GATAD2B in promoting chemoresistance, we analyzed the expression of GATAD2B in breast cancer patients receiving chemotherapy. In TNBC patients that did not respond to chemotherapy, the expression of GATAD2B was significantly higher compared to that of responding group with an area under the curve greater than 0.6 [32] (Figure 7A). This result suggests that GATAD2B may serve as a potential prognostic marker for chemoresistance in TNBC patients. To test this idea further, we stably overexpressed GATAD2B in MDA-MB-231 cells and treated with increasing dose of paclitaxel, a first line drug for TNBC [45]. In a dose-dependent manner, paclitaxel reduced the ability of MDA-MB-231 cells to grow in clonogenic assay (Figure 7B), as well as increased apoptosis (Figure 7C). However, MDA-MB-231 cells overexpressing GATAD2B are significantly resistant to paclitaxel effects compared to control cells (Figure 7B), especially at 1 nM and 2 nM. Similarly, the population of cancer cells undergoing apoptosis with GATAD2B overexpression was significantly reduced compared to control cells (Figure 7D). Similar results are seen in SUM159 cells overexpressing GATAD2B when compared to control cells (Supplementary Figure S6A,B), further confirming the potential role of GATAD2B in protecting breast cancer cells from chemotherapy. In addition, we show that overexpression of GATAD2B, but not GATAD2B-Mut, protects from paclitaxel mediated cell death (Supplementary Figure S7A,B) in MDA-MB-231 cells. These findings suggest a potential role of GATAD2B in promoting chemoresistance of breast cancer cells in vitro, and role of GATAD2B as a potential predictive marker for chemoresistance in aggressive breast cancers.

## 4. Discussion

The development of breast cancer is heavily influenced by signals from the tumor microenvironment, including the nutrient status, which is sensed by nutrient sensing mechanisms to modulate cellular activities [9]. One such mechanism is the hexosamine biosynthetic pathway, which utilizes glucose and other substrates from major metabolic pathways to produce UDP-GlcNAc [9]. This nucleotide sugar serves as a substrate for extensive N-linked and O-linked glycosylation, or for O-GlcNAcylation of intracellular proteins [9,10,12]. The process of O-GlcNAcylation and its enzyme OGT function to couple metabolism to signaling transduction in cancer cells [12]. Increased OGT and O-GlcNAc are elevated in various cancers, including breast cancer. Elevated levels of OGT and O-GlcNAc in breast cancer promote breast tumor growth and metastasis [46,47]. Recent studies have shown that OGT and O-GlcNAc play a critical role in maintaining and driving tumor initiation and CSCs phenotypes. Elevated expression of OGT increases mammosphere formation, CSCs population and expression of CSCs markers [17]. However, the mechanism of how OGT and O-GlcNAc control CSCs maintenance and function is still unclear. Here, using a global proteomic analysis, we identified GATAD2B of the NuRD complex as a target of OGT and O-GlcNAc in breast cancer. The expression of GATAD2B is critical and sufficient to maintain and promote CSCs phenotype of breast cancer cells. GATAD2B also regulates expression of CSCs markers, including Oct4, SOX2, c-Myc and NANOG, at both the mRNA and protein level. We also confirmed that GATAD2B is a critical downstream effector of OGT and O-GlcNAc in controlling the CSCs properties. In this mechanism, direct O-GlcNAcylation of GATAD2B protects from ubiquitination and proteasomal degradation, thus enhancing the protein stability. In addition, we identify ITCH as a E3 ligase that interacts with GATAD2B and regulates it degradation. This interaction between GATAD2B and ITCH is enhanced when the enzymatic activity of OGT is inhibited. ITCH knockdown increased the level of GATAD2B, but also elevated the mammosphere formation of breast cancer cells and increased cancer stem cell factors SOX2 and c-Myc. Importantly, genetic inhibition of ITCH was also able to rescue the level of GATAD2B and mammosphere formation in the presence of OGT inhibitor, which further indicated the critical role of OGT in regulating GATAD2B level to drive CSCs function. Moreover, we for the first time showed that O-GlcNAcylation within the C-terminus of GATAD2B is critical for GATAD2B stability, as well as for the CSCs properties of breast cancer cells. Other groups have shown that GATAD2B can be O-GlcNAcylated at other sites [48]. However, the functional role of these other GATAD2B O-GlcNAcylation sites need to be furthered examined. Collectively, we showed a novel regulatory mechanism of GATAD2B by OGT, which is pivotal for CSCs maintenance and function in breast cancer (Figure 7E).

GATAD2B is a component of the NuRD complex, and functions as a scaffolding protein for NuRD complex assembly [49]. Dysregulation of GATAD2B was reported in multiple neurological disorders, which is potentially linked to the role of GATAD2B and NuRD complex in regulating function of stem cells and progenitor cells [50,51]. In addition, our study shows that GATAD2B is highly upregulated in breast cancer cells and in patient samples. However, the role of GATAD2B in breast cancer has not yet been reported. We for the first time showed that GATAD2B is critical for CSCs function in breast cancer and is regulated directly by OGT via O-GlcNAcylation. This role of GATAD2B in CSCs aligns with the reported role of GATAD2B in promoting metastasis in KRAS-mutant lung cancer, strengthening the role of GATAD2B in cancer [21]. The regulation GATAD2B O-GlcNAcylation possibly results in changes in NuRD complex formation, which subsequently controls gene expression by remodeling chromatin. Although GATAD2B has an analog, GATAD2A in the NuRD complex, recent evidence suggests that NuRD complexes containing GATAD2B as opposed to GATAD2A may possess distinct functions [20,50,52]. Consistent with this idea, our results show that GATAD2B, but not GATAD2A, can regulate CSCs function in breast cancer further implicating a specific role of GATAD2B in CSCs. It will be interesting to identify in the future the specific NuRD complex bound to GATAD2B that regulates CSC functions.

O-GlcNAcylation of target protein alters the protein stability, localization, and protein-protein interaction, thus facilitating the modulation of protein functions [12]. O-GlcNAcylation on GATAD2B increases the half-life of the NuRD complex component by impairing the interaction between GATAD2B and its E3-ligase, ITCH. ITCH promotes GATAD2B ubiquitination and subsequently its proteasomal degradation. Genetic inhibition of ITCH enhances the stability of GATAD2B, mammosphere formation and expression of CSCs factors in breast cancer cells. Importantly, inhibition of OGT enzymatic activity enhances GATAD2B and ITCH interaction reducing GATAD2B level and mammosphere formation. This reduction in GATAD2B and mammosphere formation is reversed by ITCH knockdown, implicating the importance of O-GlcNAcylation on GATAD2B stability and its function in promoting CSCs. Similarly, reducing O-GlcNAcylation on GATAD2B by mutating potential modification Serines to Alanines also shortens GATAD2B half-life and increases ubiquitination level of GATAD2B, subsequently impairs mammosphere formation of breast cancer cells. This finding, for the first time, points to the novel regulating mechanism of OGT on GATAD2B via modulating GATAD2B degradation in an ITCH-dependent manner.

The E3-ligase ITCH plays a crucial role during the development of breast tumors. In breast cancer, inhibited expression of ITCH led to an increase in the activity of the Wnt/β-catenin signaling promoting the growth of breast cancer in vitro and in vivo [53]. In contrast, increased expression of ITCH resulted in decreased signaling activity of the Wnt/β-catenin, reduced cell proliferation and tumor growth [53]. In stem cells, ITCH negatively regulates the function and homeostasis of hemopoietic stem cells, where reduced expression of ITCH led to increased expression of pro-self-renewing factor Notch1, increased proliferation and sustained function of progenitor cells [54]. In addition, ITCH functions as an E3-ligase of Oct4, a key transcription factor for maintaining self-renewal, to contribute to regulate the fate of embryonic stem cells [55]. However, the potential role of ITCH in regulating cancer stem-like cells properties has not been reported. Here, we showed that genetic inhibition of ITCH increased expression of GATAD2B, SOX2 and MYC, as well as mammosphere formation and CSCs population detected by ALDEFLOUR in multiple breast cancer cell lines. The function of ITCH in regulating CSCs in breast cancer could be, in part, a result of ITCH regulation on GATAD2B stability. Together, these data show a crucial role of ITCH in mediating GATAD2B levels and regulating CSCs in breast cancer. One important characteristic of CSCs is the potential in promoting resistance to anti-cancer therapies [44]. Overexpression of GATAD2B increases the CSCs properties in breast cancer cells, but also promotes resistance to apoptosis induced by paclitaxel in vitro. Increased expression of GATAD2B in breast cancer cells protects from paclitaxel-mediated apoptosis and clonogenic survival and requires O-GlcNAcylation of GATAD2B. These findings, together with high expression of GATAD2B in breast cancer patients that are non-responsive to chemotherapy, suggests the potential of GATAD2B to serve as a clinical prognosis marker for breast cancer patients.

## 5. Conclusions

Collectively, these data suggests that O-GlcNAcylation of GATAD2B can regulate its ubiquitination and interaction with E3 ligase ITCH and thus contribute to O-GlcNAc-mediated regulation of cancer stem cell phenotypes and chemoresistance in breast cancer cells.

## Data Availability

All data included in this manuscript or in Supplementary Materials are available upon request. The mass spectrometry proteomics data have been deposited to the ProteomeXchange Consortium via the PRIDE partner repository with the dataset identifier PXD058744.

## Supplementary Materials

The following supporting information can be downloaded at: https://www.mdpi.com/article/10.3390/cells14060398/s1, Figure S1: GATAD2B is overexpressed in breast cancer and is associated with poor outcome of breast cancer patients. Figure S2: GATAD2B is critical to maintain CSCs phenotype in breast cancer cells. Figure S3: GATAD2B promotes CSCs phenotype downstream of OGT in breast cancer cells. Figure S4: GATAD2B protein levels are regulated by ITCH in breast cancer cells. Figure S5: GATAD2B O-GlcNAcylation is critical to promote CSC phenotype in breast cancer. Figure S6: GATAD2B promotes resistance to paclitaxel in breast cancer cells in vitro. Figure S7: GATAD2B O-GlcNAcylation is critical to promote resistance to paclitaxel in breast cancer cells in vitro. Table S1: Proteomic data of TNBC cells with altered O-GlcNAc level. Table S2: O-GlcNAc modified peptides detected in TNBC cells. Table S3: Proteomic data showing protein interacting with GATAD2B in TNBC cells. Table S4: Proteomic data showing O-GlcNAc modification of GATAD2B.

## References

[1] Giaquinto A.N., Sung H., Miller K.D., Kramer J.L., Newman L.A., Minihan A., Jemal A., Siegel R.L. (2022). Breast Cancer Statistics, 2022. CA Cancer J. Clin..

[2] Sousa B., Ribeiro A.S., Paredes J. (2019). Heterogeneity and Plasticity of Breast Cancer Stem Cells. Adv. Exp. Med. Biol..

[3] Celia-Terrassa T., Jolly M.K. (2020). Cancer Stem Cells and Epithelial-to-Mesenchymal Transition in Cancer Metastasis. Cold Spring Harb. Perspect. Med..

[4] Liang Y., Zhang H., Song X., Yang Q. (2020). Metastatic heterogeneity of breast cancer: Molecular mechanism and potential therapeutic targets. Semin. Cancer Biol..

[5] De Angelis M.L., Francescangeli F., Zeuner A. (2019). Breast Cancer Stem Cells as Drivers of Tumor Chemoresistance, Dormancy and Relapse: New Challenges and Therapeutic Opportunities. Cancers.

[6] Zheng Q., Zhang M., Zhou F., Zhang L., Meng X. (2020). The Breast Cancer Stem Cells Traits and Drug Resistance. Front. Pharmacol..

[7] Zhou H.M., Zhang J.G., Zhang X., Li Q. (2021). Targeting cancer stem cells for reversing therapy resistance: Mechanism, signaling, and prospective agents. Signal Transduct. Target. Ther..

[8] Dupuy F., Tabaries S., Andrzejewski S., Dong Z., Blagih J., Annis M.G., Omeroglu A., Gao D., Leung S., Amir E. (2015). PDK1-Dependent Metabolic Reprogramming Dictates Metastatic Potential in Breast Cancer. Cell Metab..

[9] Akella N.M., Ciraku L., Reginato M.J. (2019). Fueling the fire: Emerging role of the hexosamine biosynthetic pathway in cancer. BMC Biol..

[10] de Queiroz R.M., Oliveira I.A., Piva B., Bouchuid Catao F., da Costa Rodrigues B., da Costa Pascoal A., Diaz B.L., Todeschini A.R., Caarls M.B., Dias W.B. (2019). Hexosamine Biosynthetic Pathway and Glycosylation Regulate Cell Migration in Melanoma Cells. Front. Oncol..

[11] Sunden M., Upadhyay D., Banerjee R., Sipari N., Fellman V., Kallijarvi J., Purhonen J. (2023). Enzymatic assay for UDP-GlcNAc and its application in the parallel assessment of substrate availability and protein O-GlcNAcylation. Cell Rep. Methods.

[12] Ciraku L., Esquea E.M., Reginato M.J. (2022). O-GlcNAcylation regulation of cellular signaling in cancer. Cell Signal.

[13] Nie H., Ju H., Fan J., Shi X., Cheng Y., Cang X., Zheng Z., Duan X., Yi W. (2020). O-GlcNAcylation of PGK1 coordinates glycolysis and TCA cycle to promote tumor growth. Nat. Commun..

[14] Zhu Q., Zhou H., Wu L., Lai Z., Geng D., Yang W., Zhang J., Fan Z., Qin W., Wang Y. (2022). O-GlcNAcylation promotes pancreatic tumor growth by regulating malate dehydrogenase 1. Nat. Chem. Biol..

[15] Jiang M., Xu B., Li X., Shang Y., Chu Y., Wang W., Chen D., Wu N., Hu S., Zhang S. (2019). O-GlcNAcylation promotes colorectal cancer metastasis via the miR-101-O-GlcNAc/EZH2 regulatory feedback circuit. Oncogene.

[16] Wu D., Jin J., Qiu Z., Liu D., Luo H. (2020). Functional Analysis of O-GlcNAcylation in Cancer Metastasis. Front. Oncol..

[17] Akella N.M., Le Minh G., Ciraku L., Mukherjee A., Bacigalupa Z.A., Mukhopadhyay D., Sodi V.L., Reginato M.J. (2020). O-GlcNAc Transferase Regulates Cancer Stem-like Potential of Breast Cancer Cells. Mol. Cancer Res..

[18] Denslow S.A., Wade P.A. (2007). The human Mi-2/NuRD complex and gene regulation. Oncogene.

[19] Schmolka N., Karemaker I.D., Cardoso da Silva R., Recchia D.C., Spegg V., Bhaskaran J., Teske M., de Wagenaar N.P., Altmeyer M., Baubec T. (2023). Dissecting the roles of MBD2 isoforms and domains in regulating NuRD complex function during cellular differentiation. Nat. Commun..

[20] Wang B., Li C., Ming J., Wu L., Fang S., Huang Y., Lin L., Liu H., Kuang J., Zhao C. (2023). The NuRD complex cooperates with SALL4 to orchestrate reprogramming. Nat. Commun..

[21] Grzeskowiak C.L., Kundu S.T., Mo X., Ivanov A.A., Zagorodna O., Lu H., Chapple R.H., Tsang Y.H., Moreno D., Mosqueda M. (2018). In vivo screening identifies GATAD2B as a metastasis driver in KRAS-driven lung cancer. Nat. Commun..

[22] Lai A.Y., Wade P.A. (2011). Cancer biology and NuRD: A multifaceted chromatin remodelling complex. Nat. Rev. Cancer.

[23] Tang B., Raviv A., Esposito D., Flanders K.C., Daniel C., Nghiem B.T., Garfield S., Lim L., Mannan P., Robles A.I. (2015). A flexible reporter system for direct observation and isolation of cancer stem cells. Stem Cell Rep..

[24] Burt R.A., Dejanovic B., Peckham H.J., Lee K.A., Li X., Ounadjela J.R., Rao A., Malaker S.A., Carr S.A., Myers S.A. (2021). Novel Antibodies for the Simple and Efficient Enrichment of Native O-GlcNAc Modified Peptides. Mol. Cell Proteom..

[25] Hou C., Deng J., Wu C., Zhang J., Byers S., Moremen K.W., Pei H., Ma J. (2024). Ultradeep O-GlcNAc proteomics reveals widespread O-GlcNAcylation on tyrosine residues of proteins. Proc. Natl. Acad. Sci. USA.

[26] Zhao P., Viner R., Teo C.F., Boons G.J., Horn D., Wells L. (2011). Combining high-energy C-trap dissociation and electron transfer dissociation for protein O-GlcNAc modification site assignment. J. Proteome Res..

[27] Maynard J.C., Chalkley R.J. (2021). Methods for Enrichment and Assignment of N-Acetylglucosamine Modification Sites. Mol. Cell Proteomics.

[28] Cerami E., Gao J., Dogrusoz U., Gross B.E., Sumer S.O., Aksoy B.A., Jacobsen A., Byrne C.J., Heuer M.L., Larsson E. (2012). The cBio cancer genomics portal: An open platform for exploring multidimensional cancer genomics data. Cancer Discov..

[29] Gao J., Aksoy B.A., Dogrusoz U., Dresdner G., Gross B., Sumer S.O., Sun Y., Jacobsen A., Sinha R., Larsson E. (2013). Integrative analysis of complex cancer genomics and clinical profiles using the cBioPortal. Sci. Signal.

[30] Chandrashekar D.S., Karthikeyan S.K., Korla P.K., Patel H., Shovon A.R., Athar M., Netto G.J., Qin Z.S., Kumar S., Manne U. (2022). UALCAN: An update to the integrated cancer data analysis platform. Neoplasia.

[31] Gyorffy B. (2021). Survival analysis across the entire transcriptome identifies biomarkers with the highest prognostic power in breast cancer. Comput. Struct. Biotechnol. J..

[32] Fekete J.T., Gyorffy B. (2019). ROCplot.org: Validating predictive biomarkers of chemotherapy/hormonal therapy/anti-HER2 therapy using transcriptomic data of 3,104 breast cancer patients. Int. J. Cancer.

[33] Fuentes-Garcia G., Castaneda-Patlan M.C., Vercoutter-Edouart A.S., Lefebvre T., Robles-Flores M. (2019). O-GlcNAcylation Is Involved in the Regulation of Stem Cell Markers Expression in Colon Cancer Cells. Front. Endocrinol..

[34] Le Minh G., Reginato M.J. (2023). Role of O-GlcNAcylation on cancer stem cells: Connecting nutrient sensing to cell plasticity. Adv. Cancer Res..

[35] Ge S.X., Jung D., Yao R. (2020). ShinyGO: A graphical gene-set enrichment tool for animals and plants. Bioinformatics.

[36] Hu G., Wade P.A. (2012). NuRD and pluripotency: A complex balancing act. Cell Stem Cell.

[37] Ponti D., Costa A., Zaffaroni N., Pratesi G., Petrangolini G., Coradini D., Pilotti S., Pierotti M.A., Daidone M.G. (2005). Isolation and in vitro propagation of tumorigenic breast cancer cells with stem/progenitor cell properties. Cancer Res..

[38] Lombardo Y., de Giorgio A., Coombes C.R., Stebbing J., Castellano L. (2015). Mammosphere formation assay from human breast cancer tissues and cell lines. J. Vis. Exp..

[39] Shaw F.L., Harrison H., Spence K., Ablett M.P., Simoes B.M., Farnie G., Clarke R.B. (2012). A detailed mammosphere assay protocol for the quantification of breast stem cell activity. J. Mammary Gland. Biol. Neoplasia.

[40] Marcato P., Dean C.A., Giacomantonio C.A., Lee P.W. (2011). Aldehyde dehydrogenase: Its role as a cancer stem cell marker comes down to the specific isoform. Cell Cycle.

[41] Das B., Pal B., Bhuyan R., Li H., Sarma A., Gayan S., Talukdar J., Sandhya S., Bhuyan S., Gogoi G. (2019). MYC Regulates the HIF2alpha Stemness Pathway via Nanog and Sox2 to Maintain Self-Renewal in Cancer Stem Cells versus Non-Stem Cancer Cells. Cancer Res..

[42] Bourguignon L.Y., Wong G., Earle C., Chen L. (2012). Hyaluronan-CD44v3 interaction with Oct4-Sox2-Nanog promotes miR-302 expression leading to self-renewal, clonal formation, and cisplatin resistance in cancer stem cells from head and neck squamous cell carcinoma. J. Biol. Chem..

[43] Wang Z., Udeshi N.D., Slawson C., Compton P.D., Sakabe K., Cheung W.D., Shabanowitz J., Hunt D.F., Hart G.W. (2010). Extensive crosstalk between O-GlcNAcylation and phosphorylation regulates cytokinesis. Sci. Signal.

[44] Phi L.T.H., Sari I.N., Yang Y.G., Lee S.H., Jun N., Kim K.S., Lee Y.K., Kwon H.Y. (2018). Cancer Stem Cells (CSCs) in Drug Resistance and their Therapeutic Implications in Cancer Treatment. Stem Cells Int..

[45] Schmid P., Abraham J., Chan S., Wheatley D., Brunt A.M., Nemsadze G., Baird R.D., Park Y.H., Hall P.S., Perren T. (2020). Capivasertib Plus Paclitaxel Versus Placebo Plus Paclitaxel As First-Line Therapy for Metastatic Triple-Negative Breast Cancer: The PAKT Trial. J. Clin. Oncol..

[46] Ferrer C.M., Lu T.Y., Bacigalupa Z.A., Katsetos C.D., Sinclair D.A., Reginato M.J. (2017). O-GlcNAcylation regulates breast cancer metastasis via SIRT1 modulation of FOXM1 pathway. Oncogene.

[47] Krzeslak A., Forma E., Bernaciak M., Romanowicz H., Brys M. (2012). Gene expression of O-GlcNAc cycling enzymes in human breast cancers. Clin. Exp. Med..

[48] Wulff-Fuentes E., Berendt R.R., Massman L., Danner L., Malard F., Vora J., Kahsay R., Olivier-Van Stichelen S. (2021). The human O-GlcNAcome database and meta-analysis. Sci. Data.

[49] Pierson T.M., Otero M.G., Grand K., Choi A., Graham J.M., Young J.I., Mackay J.P. (2019). The NuRD complex and macrocephaly associated neurodevelopmental disorders. Am. J. Med. Genet. C Semin. Med. Genet..

[50] Xing G., Liu Z., Huang L., Zhao D., Wang T., Yuan H., Wu Y., Li L., Long Q., Zhou Y. (2022). MAP2K6 remodels chromatin and facilitates reprogramming by activating Gatad2b-phosphorylation dependent heterochromatin loosening. Cell Death Differ..

[51] Shieh C., Jones N., Vanle B., Au M., Huang A.Y., Silva A.P.G., Lee H., Douine E.D., Otero M.G., Choi A. (2020). GATAD2B-associated neurodevelopmental disorder (GAND): Clinical and molecular insights into a NuRD-related disorder. Genet. Med..

[52] Mor N., Rais Y., Sheban D., Peles S., Aguilera-Castrejon A., Zviran A., Elinger D., Viukov S., Geula S., Krupalnik V. (2018). Neutralizing Gatad2a-Chd4-Mbd3/NuRD Complex Facilitates Deterministic Induction of Naive Pluripotency. Cell Stem Cell.

[53] Lim S.K., Lu S.Y., Kang S.A., Tan H.J., Li Z., Adrian Wee Z.N., Guan J.S., Reddy Chichili V.P., Sivaraman J., Putti T. (2016). Wnt Signaling Promotes Breast Cancer by Blocking ITCH-Mediated Degradation of YAP/TAZ Transcriptional Coactivator WBP2. Cancer Res..

[54] Rathinam C., Matesic L.E., Flavell R.A. (2011). The E3 ligase Itch is a negative regulator of the homeostasis and function of hematopoietic stem cells. Nat. Immunol..

[55] Liao B., Zhong X., Xu H., Xiao F., Fang Z., Gu J., Chen Y., Zhao Y., Jin Y. (2013). Itch, an E3 ligase of Oct4, is required for embryonic stem cell self-renewal and pluripotency induction. J. Cell Physiol..

