# Supplementary material for: GATAD2B O-GlcNAcylation Regulates Breast Cancer Stem-like Potential and Drug Resistance
Source: Cells. 2025 Mar 8;14(6):398. doi: 10.3390/cells14060398 (PMC11941746; doi:10.3390/cells14060398)
Supplement: Supplementary file 1 [file cells-14-00398-s001.zip › Supplemental Figure Legends.pdf]

Supplemental Figures.

Supplemental Figure S1: GATAD2B is overexpressed in breast cancer and is associated with poor outcome of breast cancer patients. (A) – TNBC cells MDA-MB-231 were treated with DMSO or OGA inhibitor Thiamet-G (2µM) for 24h and were subsequently collected for Western blot using indicated antibodies (left). Quantified graph shows increased level of NuRD complex components in the presence of Thiamet-G compared to DMSO (right). (B) – OncoPrint of NuRD complex components was generated by cBioportal, METABRIC database. OncoPrint data shows differential dysregulation of NuRD complex components. (C) – Database analysis shows an increased level of GATAD2B in major subclasses of breast cancer, compared to normal tissue. (D) – Kaplan-Meier plot showing differential survival rate of breast cancer patients with various levels of GATAD2B mRNA (auto select best cutoff, overall survival, PAM50 subtype: basal). (E) – Kaplan-Meier plots show differential survival rate of breast cancer with various levels of GATAD2B protein (auto select best cutoff, overall survival, Tang_2018). (F) – TNBC SUM159 cells, control or overexpressing OGT, were collected for immunoblot using indicated antibodies (top). Quantified graph showing GATAD2B level detected by immunoblot in SUM159 cells, control or overexpressing OGT (bottom). One sample t-test against hypothetical value is reported as mean ± SEM, *p<0.05. (G) – TNBC SUM159 cells, transduced with scramble or OGT shRNA, were collected for immunoblot using indicated antibodies (top). Quantified graph showing GATAD2B level detected by immunoblot in SUM159 cells, transduced with scramble or OGT shRNA (bottom). One sample t-test against hypothetical value is reported as mean ± SEM, **p<0.01.

Supplemental Figure S2: GATAD2B is critical to maintain CSCs phenotype in breast cancer cells. (A) – TNBC cells MDA-MB-231, SUM159 and PDX cells HCI-10 were grown in monolayer or in mammospheres and were subsequently collected for Western blot using indicated antibodies (left). Quantified graph shows increased level of GATAD2B in mammosphere compared to control cells in monolayer (right). Multiple one sample t-test against hypothetical value with Holm-Sidak correction is reported as mean ± SEM, *p<0.05. (B) – TNBC SUM159 cells were transduced with control (scramble) or with GATAD2B-specific shRNAs. Lysates from control or transduced with GATAD2B shRNAs SUM159 cells growing in monolayer (left, top) or in mammosphere (left, bottom) were collected for immunoblot analysis using indicated antibodies. Control or transduced with GATAD2B shRNAs SUM159 cells were allowed to grow in mammosphere formation assay. Mammospheres were counted and primary mammosphere formation efficiency from each condition was determined and graphed (right). Primary mammospheres were collected and regrown in the same mammosphere culture condition to form secondary mammosphere. The number of mammospheres and secondary mammosphere formation efficiency from each condition was determined and graph (right). Two-way ANOVA with Sidak test is reported as mean ± SEM, *p<0.05. (C) – PDX cells HCI-10 were transduced with control (scramble) or with GATAD2B-specific shRNAs. Lysates from control or transduced with GATAD2B shRNAs HCI-10 cells growing in monolayer (left, top) or in mammosphere (left, bottom) were collected for immunoblot analysis using indicated antibodies. Control or transduced with GATAD2B shRNAs HCI-10 cells were allowed to grow in mammosphere formation assay. Mammospheres were counted and primary mammosphere formation efficiency from each condition was determined and graphed (right). Primary mammospheres were collected and regrown in the same mammosphere culture condition to form secondary mammosphere. The number of mammospheres and secondary mammosphere formation efficiency from each condition was determined and graph (right). Two-way ANOVA

with Sidak test is reported as mean ± SEM, *p<0.05, **p<0.01, ***p<0.001. (D) – Quantified graph of SORE-GFP+ CSCs in MDA-MB-231 cells transduced with scramble or GATAD2B shRNAs. One-way ANOVA with Sidak test is reported as mean ± SEM, *p<0.05. (E) – TNBC cells MDA-MB-231 transduced with scramble or GATAD2A shRNA were collected for Western blot using indicated antibodies (left). Quantified graph shows mammosphere of MDA-MB-231 cells transduced with scramble or GATAD2A shRNA (right). Paired t-test is reported as mean ± SEM, ns – not significant.

Supplemental Figure S3: GATAD2B promotes CSCs phenotype downstream of OGT in breast cancer cells. (A) – Lysates from control or GATAD2B overexpressing SUM159 cells were collected for immunoblot analysis using indicated antibodies. Control or GATAD2B overexpressing SUM159 cells were allowed to grow in mammosphere formation assay. Mammospheres were counted and mammosphere formation efficiency from each condition was determined and graphed (right). Paired t-test is reported as mean ± SEM, **p<0.01. (B) – Lysates from control or GATAD2B overexpressing HCI-10 cells were collected for immunoblot analysis using indicated antibodies. Control or GATAD2B overexpressing HCI-10 cells were allowed to grow in mammosphere formation assay. Mammospheres were counted and mammosphere formation efficiency from each condition was determined and graphed (right). Paired t-test is reported as mean ± SEM, **p<0.01. (C) – Quantified graph shows increase in the population of ALDH+ CSCs in SUM159 cells, control or overexpressing GATAD2B. Paired t-test is reported as mean ± SEM, *p<0.05. (D) – Lysates from control or OGT overexpressing SUM159 cells, transduced with scramble or GATAD2B shRNAs, were collected and analyzed by immunoblot using indicated antibodies (top). Control or OGT overexpressing SUM159 cells, transduced with scramble or GATAD2B shRNAs, were grown in mammosphere formation assay. The mammosphere formation efficiency in each condition was determined and presented in the quantified graph (bottom). Two-way ANOVA with Sidak test is reported as mean ± SEM, *p<0.05, **p<0.01. (E) – Lysates from control or transduced with GATAD2B shRNAs SUM159 cells, treated with DMSO or OGA inhibitor Thiamet-G (2µM) for 48h, were collected and analyzed by immunoblot using indicated antibodies (left). Control or transduced with GATAD2B shRNAs SUM159 cells, treated with DMSO or OGA inhibitor Thiamet-G (2µM) for 48h, were grown in mammosphere formation assay. The mammosphere formation efficiency in each condition was determined and presented in the quantified graph (right). Two-way ANOVA with Sidak test is reported as mean ± SEM, *p<0.05, **p<0.01, ***p<0.001.

Supplemental Figure S4: GATAD2B protein levels are regulated by ITCH in breast cancer cells. (A) – TNBC cells MDA-MB-231 and SUM159 were treated with DMSO or OGA inhibitor Thiamet-G (2µM) for 24h. Total RNA from each condition was collected and mRNA levels of indicated genes were analyzed by qRT-PCR. One sample t-test against hypothetical value is reported as mean ± SEM, ns – not significant. (B) – Quantified graph of 5B shows increased level of Ubiquitin co-immunoprecipitated with GATAD2B. One sample t-test against hypothetical value is reported as mean ± SEM, *p<0.05. (C) – Volcano plots shows mass spectrometry result of TNBC cells MDA-MB-231, treated with DMSO or OGA inhibitor Thiamet-G (2µM). (D) – TNBC cells MDA-MB-231 expressing control or ITCH shRNA were treated with OGT inhibitor OSMi-1 (100µM) for 24h, then were collected for immunoprecipitation using anti-GATAD2B antibody or IgG. Immunoprecipitated proteins were analyzed by Western blot using indicated antibodies (left). Quantified graph shows decrease level of Ubiquitin being co-immunoprecipitated with

GATAD2B in the presence of ITCH shRNA compared to control shRNA (right). One sample t-test against hypothetical value is reported as mean ± SEM, *p<0.05. (E) – PDX cells HCI-10 were transduced with scramble or ITCH shRNAs and were subsequently collected for immunoblot analysis using indicated antibodies (left). Control or transduced with ITCH shRNAs PDX cells HCI-10 were plated in mammosphere formation assay. Mammosphere formation efficiency from each condition was determined and graphed (right). One-way ANOVA with Sidak test is reported as mean ± SEM, *p<0.05. (F) – TNBC cells SUM159 were transduced with scramble or ITCH shRNAs and were subsequently collected for immunoblot analysis using indicated antibodies (left). Control or transduced with ITCH shRNAs TNBC cells SUM159 were plated in mammosphere formation assay. Mammosphere formation efficiency from each condition was determined and graphed (right). One-way ANOVA with Sidak test is reported as mean ± SEM, *p<0.05.

Supplemental Figure S5: GATAD2B O-GlcNAcylation is critical to promote CSC phenotype in breast cancer. (A) – PDX cells HCI-10, transduced with control or GATAD2B gRNA, were infected with lentiviral vectors containing wild-type or O-GlcNAc sites mutant GATAD2B. Cell lysates were collected and analyzed by immunoblot using indicated antibodies. (B) – Quantified graph shows mammosphere formation efficiency of control or transduced with GATAD2B gRNA HCI-10 cells overexpressing WT or Mutant GATAD2B (right). Two-way ANOVA with Sidak test is reported as mean ± SEM, ns – not significant, *p<0.05, **p<0.01.

Supplemental Figure S6: GATAD2B promotes resistance to paclitaxel in breast cancer cells *in vitro*. (A) – TNBC SUM159 cells, control or overexpressing GATAD2B, were collected for Western blot using indicated antibodies (top, left). Control or GATAD2B overexpressing TNBC cells SUM159 were treated with an increasing dose of paclitaxel for 48h. DMSO was used as control. Cells were plated in clonogenic assay and were allowed to grow for 10-14 days. Colonies were stained, counted. Representative images show stained colonies (top-right). Quantified graph shows increased number of colonies in GATAD2B overexpression compared to control cells (bottom). Two-way ANOVA with Sidak test is reported as mean ± SEM, *p<0.05. (B) – Quantified graph showing percentage of dead cells from control or GATAD2B overexpressing SUM159 cells treated with increasing dose of paclitaxel for 48h. Two-way ANOVA with Sidak test is reported as mean ± SEM, *p<0.05.

Supplemental Figure S7: GATAD2B O-GlcNAcylation is critical to promote resistance to paclitaxel in breast cancer cells *in vitro*. (A) – TNBC cells MDA-MB-231, transduced with GATAD2B gRNA, were infected with lentiviral vectors containing wild-type or O-GlcNAc sites mutant GATAD2B. Cells lysates were collected for immunoblot analysis using indicated antibodies (top-left). MDA-MB-231 cells expressing wild-type or mutant GATAD2B were treated with increasing dose of paclitaxel for 48h. DMSO was used as control. Cells were plated in clonogenic assay and were allowed to grow for 10-14 days. Colonies were stained, counted. Representative images show stained colonies (top-right). Quantified graph shows increased number of colonies in wild-type GATAD2B compared to mutant GATAD2B (bottom). Two-way ANOVA with Sidak test is reported as mean ± SEM, *p<0.05, **p<0.01. (B) – Quantified graph showing percentage of dead cells MDA-MB-231 cells expressing wild-type or mutant GATAD2B treated with increasing dose of paclitaxel for 48h. Two-way ANOVA with Sidak test is reported as mean ± SEM, *p<0.01, ***p<0.001.

Supplemental Tables.

Supplemental Table S1: Proteomic data of TNBC cells with altered O-GlcNAc level. TNBC cell MDA-MB-231 were treated with Thiamet-G (1μM) for 24h before being lysed and digested by trypsin. Digested peptides were separated and analyzed by LC-MS/MS in a label-free proteomic analysis. Sheet 1 highlights the log2 fold changes, p values, and q values of peptides abundancy in control cells and in cells treated with Thiamet-G.

Supplemental Table S2: O-GlcNAc modified peptides detected in TNBC cells. TNBC cell MDA-MB-231 were treated with Thiamet-G (1μM) for 24h before being lysed, digested with trypsin and PNGaseF. O-GlcNAcylated peptides were immunoaffinity purified and analyzed by LC-MS/MS with alternating HCD and ETD or product ion triggered ETD. Sheet 1 shows putative O-GlcNAc modified peptides observed.

Supplemental Table S3: Proteomic data showing protein interacting with GATAD2B in TNBC cells. TNBC cells treated with DMSO, Thiamet-G (10μM) or OSMi (10μM) were collected and lysed before being immunoprecipiated by anti-GATAD2B antibodies. Immunoprecipiated fraction were resolved and analyzed by LC-MS. Binary comparisons were performed between GATAD2B IP vs IgG control; GATAD2B_ThiametG IP vs IgG control; and GATAD2B_OSMI IP vs IgG control. Sheet 1 highlights the p-value of two-sample t-test and log2 fold change of peptides abundancy in cancer cells treated with DMSO, Thiamet-G or OSMi.

Supplemental Table S4: Proteomic data showing O-GlcNAc modification of GATAD2B. HEK-293T cells overexpressing GATAD2B were collected and lysed. GATAD2B were immunoprecipitated and gel-purified before being digested by trypsin. Digested peptides were resolved and analyzed by LC-MS/MS to identify O-GlcNAc sites.
