# Supplementary figures and images for: GATAD2B O-GlcNAcylation Regulates Breast Cancer Stem-like Potential and Drug Resistance
Source: Cells. 2025 Mar 8;14(6):398. doi: 10.3390/cells14060398 (PMC11941746; doi:10.3390/cells14060398)

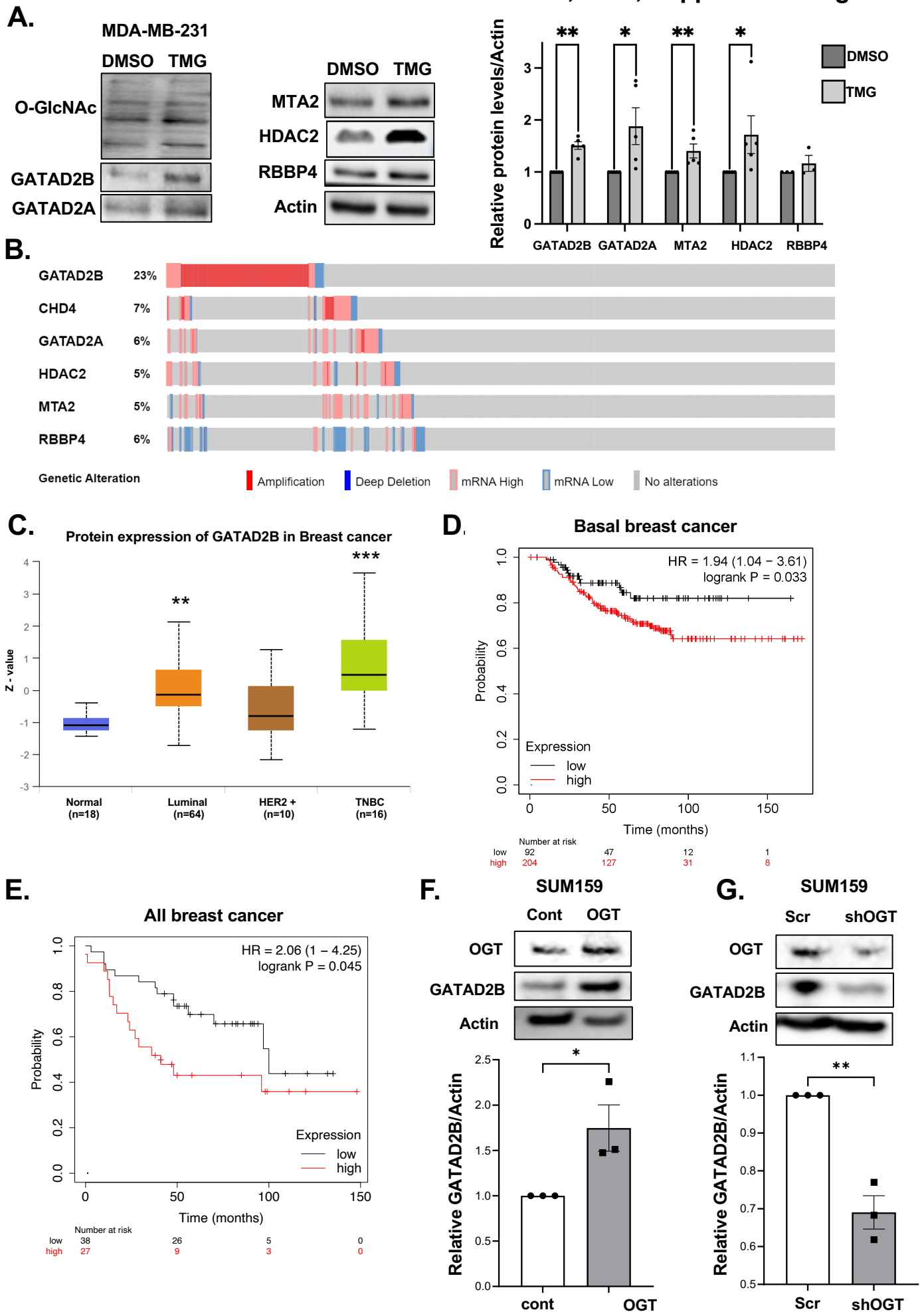

**A.**

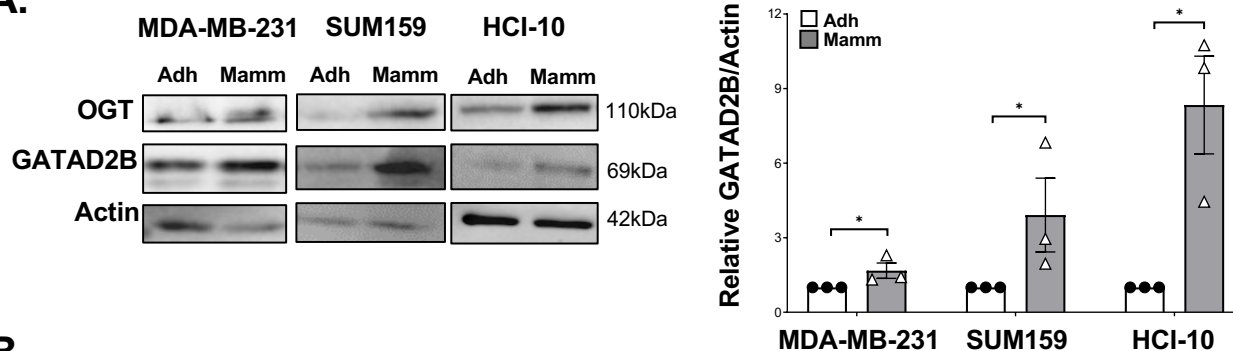

**B.**

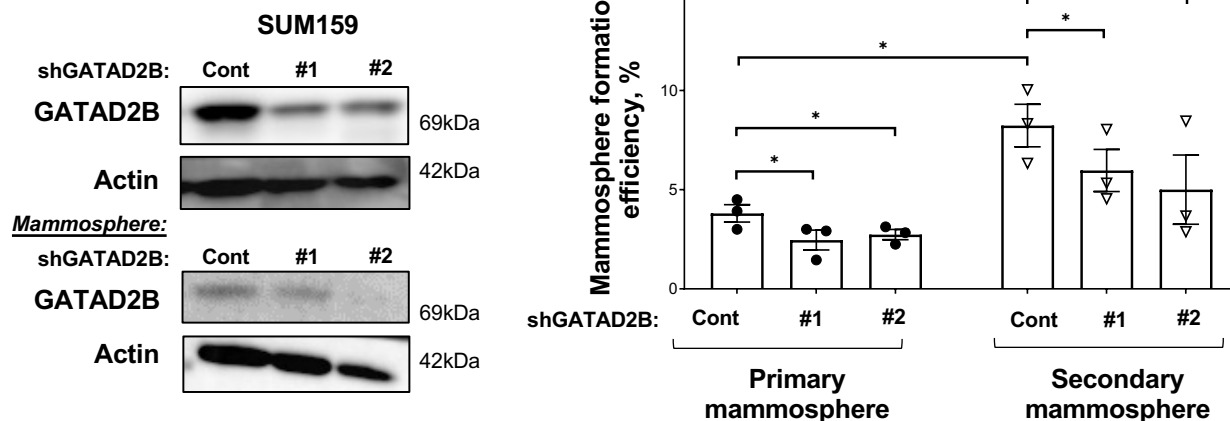

**C.**

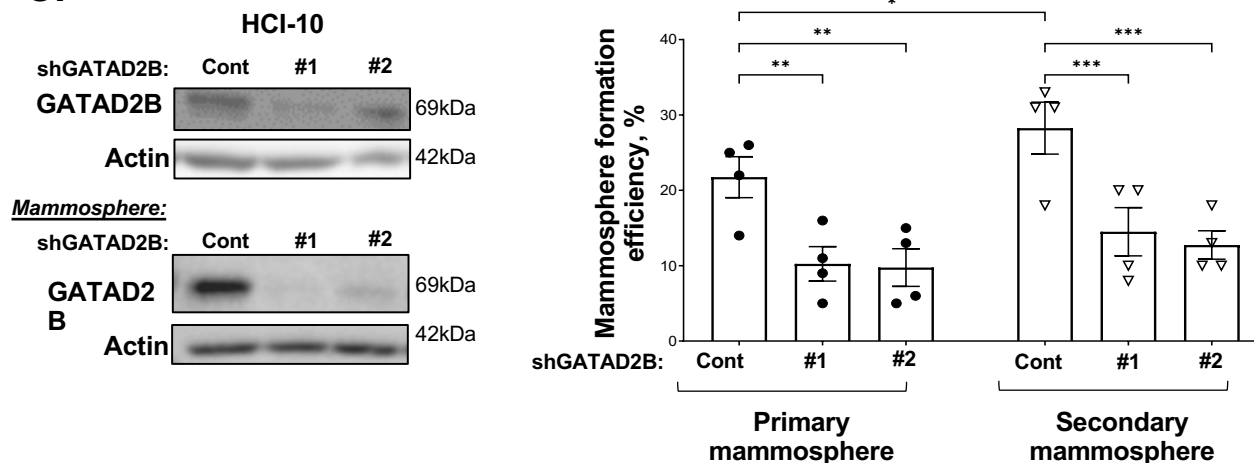

**D.**

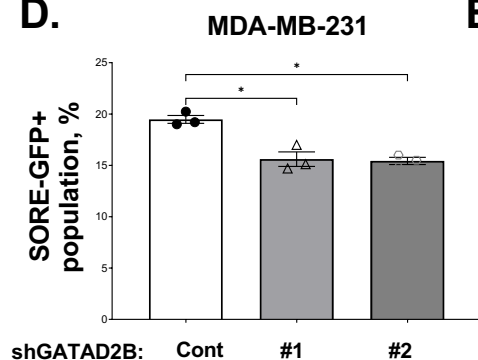

**E.**

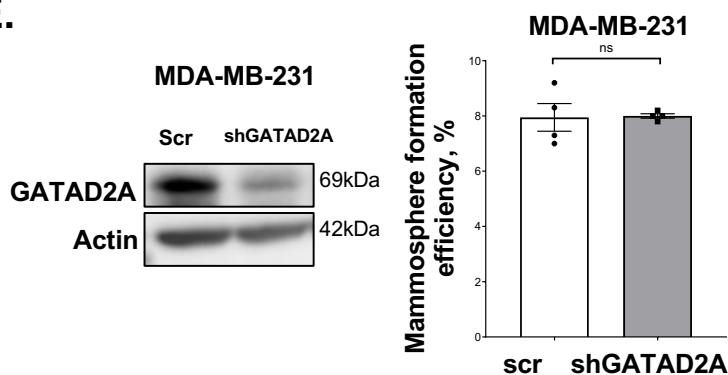

**A.**

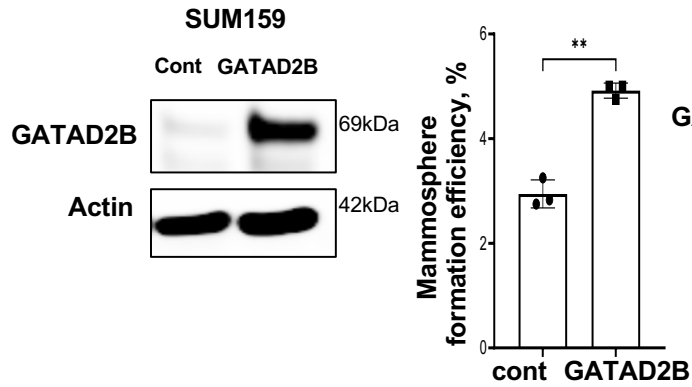

**B.**

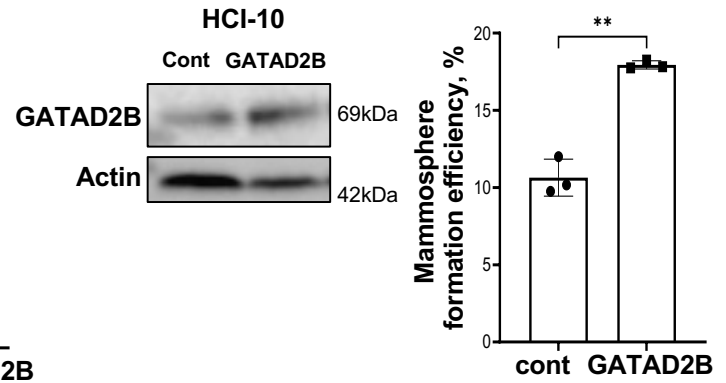

**C.**

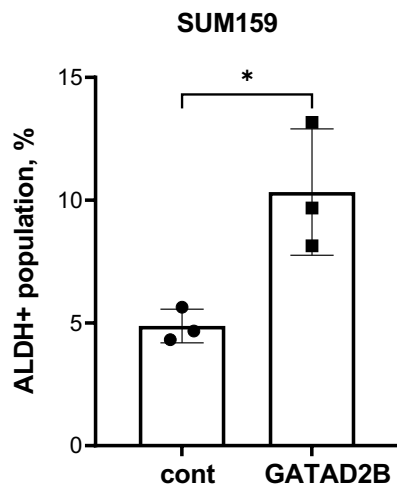

**D.**

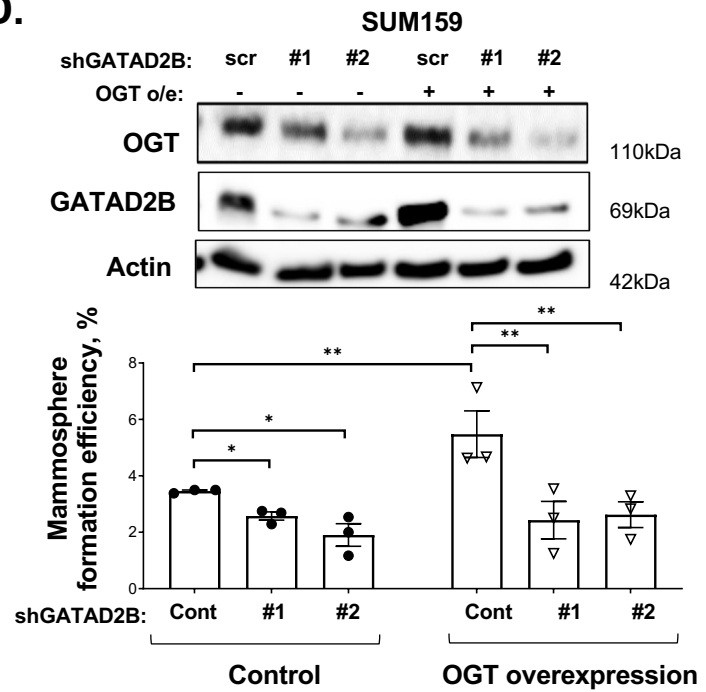

**E.**

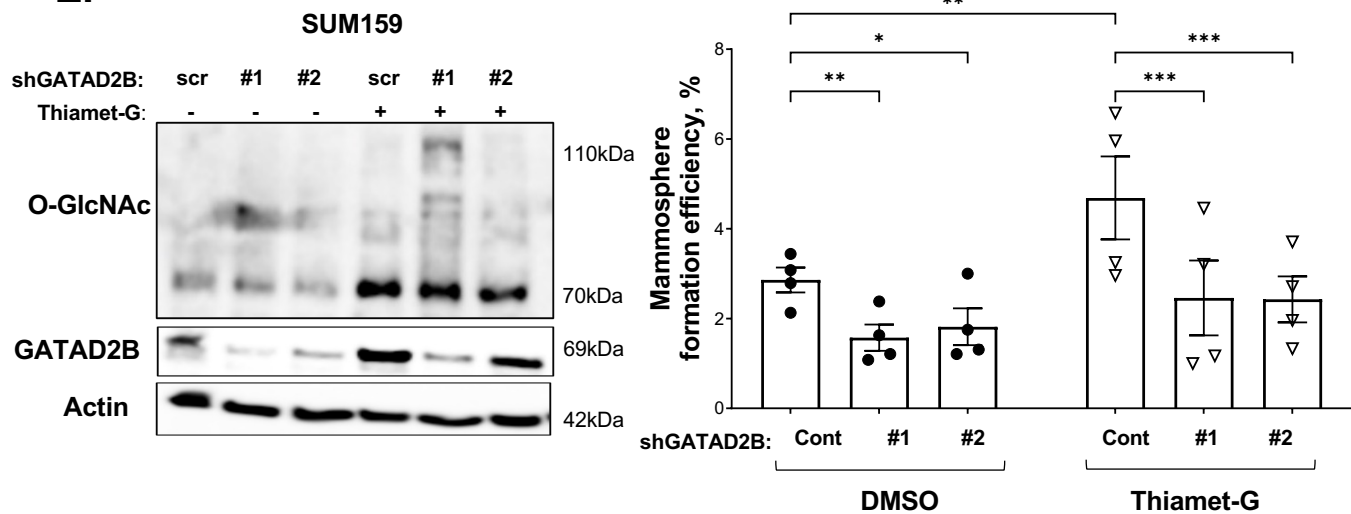

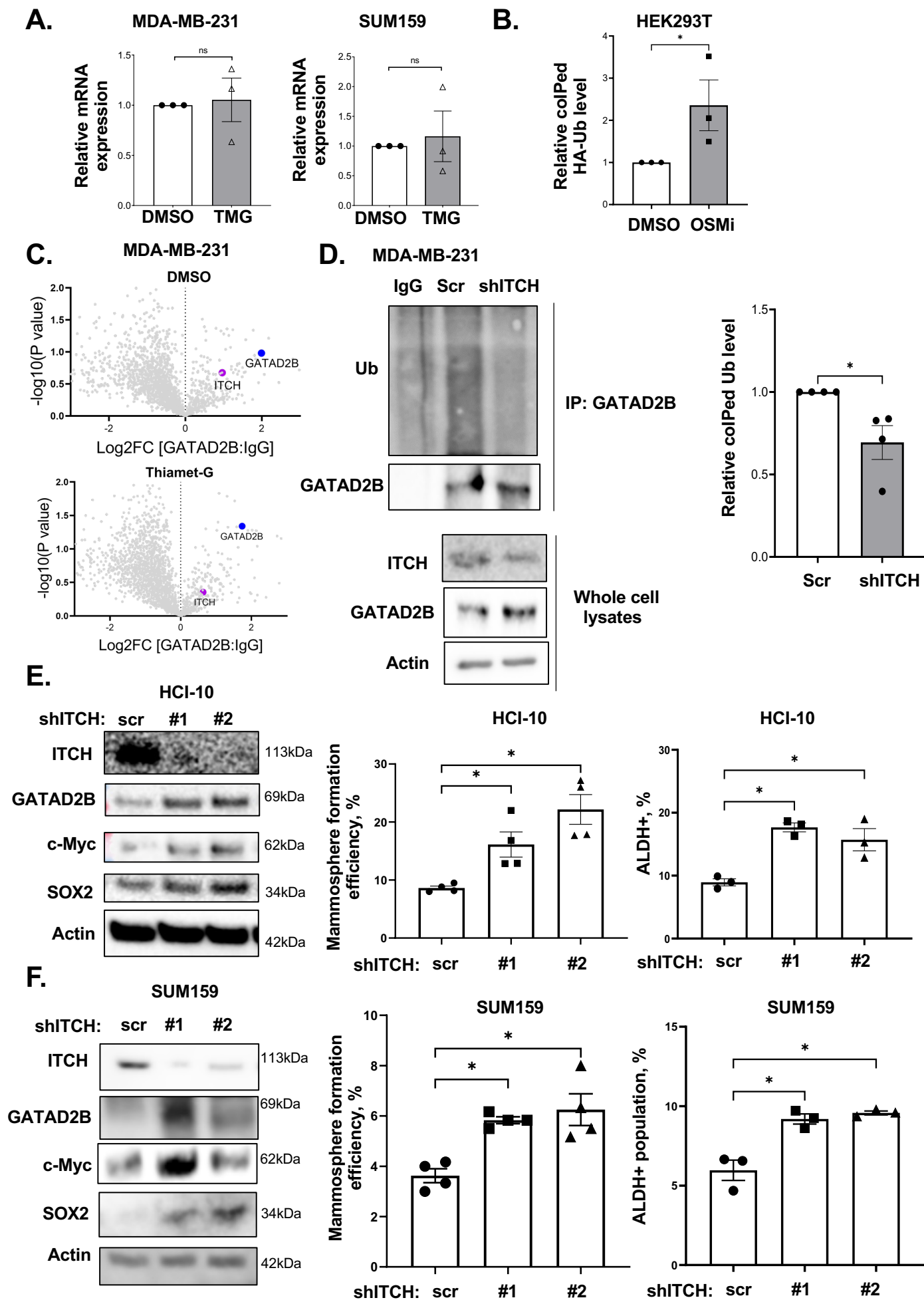

A.

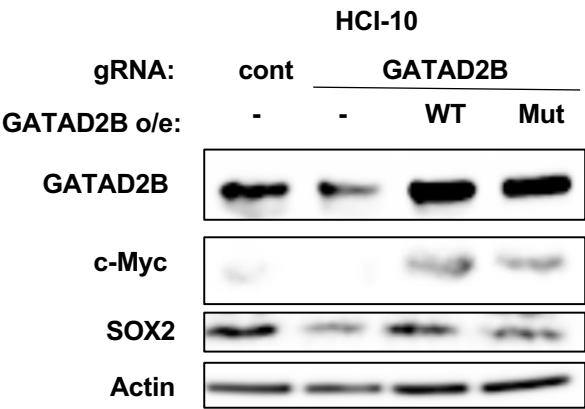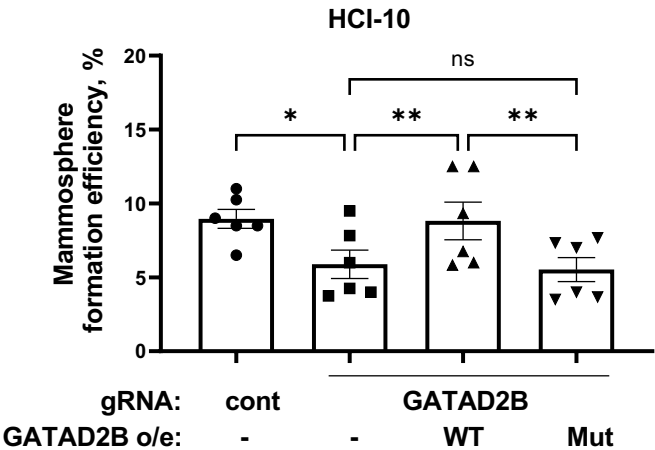

**A.**

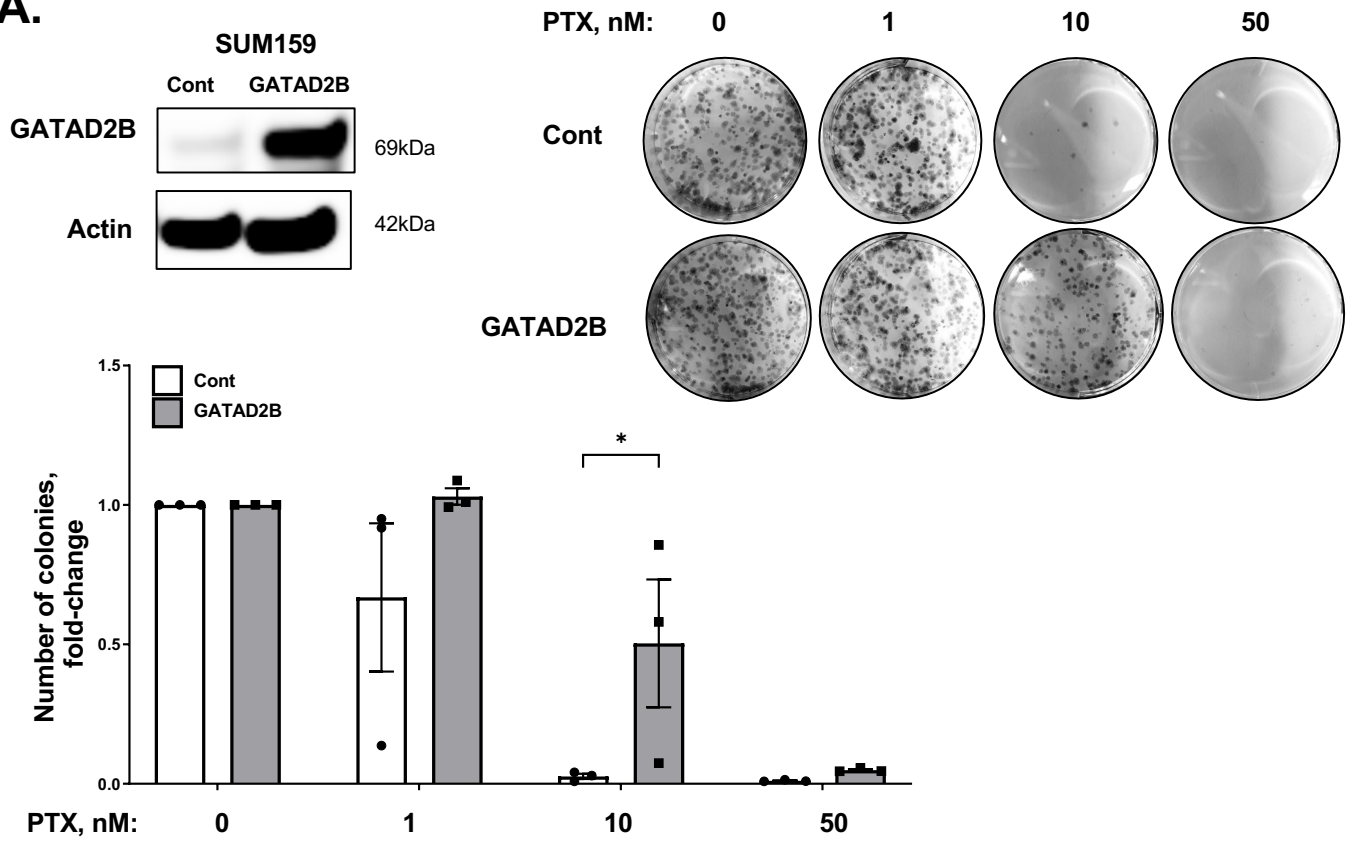

**B.**

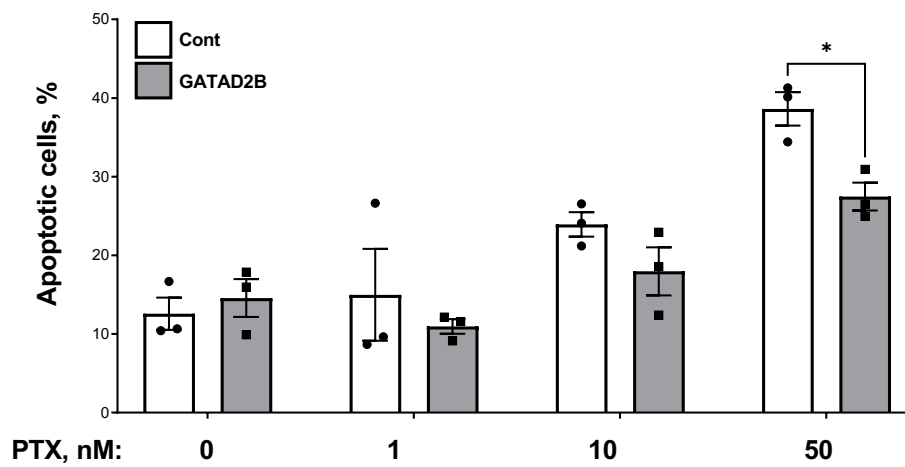

**A.**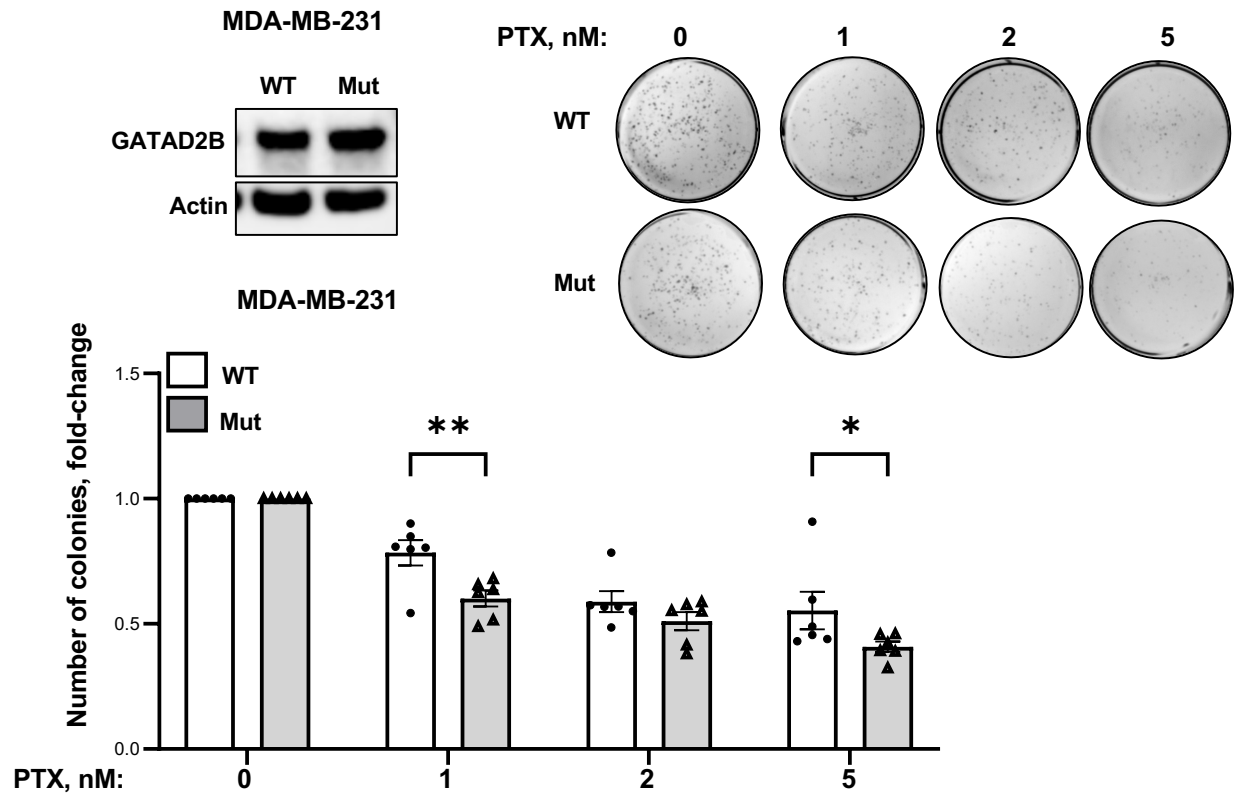**B.**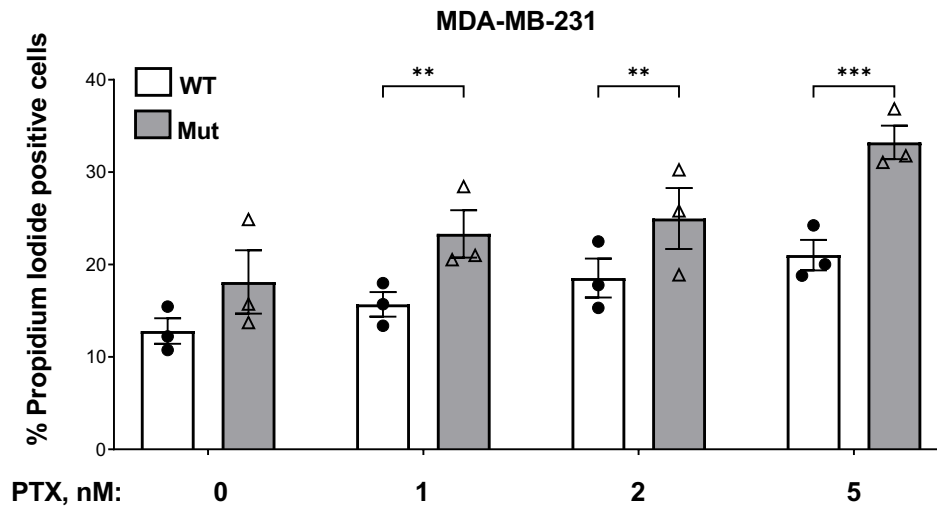

Supplement: Supplementary file 1 [file cells-14-00398-s001.zip › Le Minh.Supplemental Figs.pdf]
